# Supplementary material for: Self-supervised learning of accelerometer data provides new insights for sleep and its association with mortality
Source: medRxiv. 2023 Jul 8:2023.07.07.23292251. Preprint. [Version 1] doi: 10.1101/2023.07.07.23292251 (PMC10350137; doi:10.1101/2023.07.07.23292251)
Supplement: 1 [file NIHPP2023.07.07.23292251v1-supplement-1.pdf]

medRxiv preprint doi: https://doi.org/10.1101/2023.07.07.23292251; this version posted July 8, 2023. The copyright holder for this preprint (which was not certified by peer review) is the author/funder, who has granted medRxiv a license to display the preprint in perpetuity.
It is made available under a CC-BY 4.0 International license .

Supplements

List of Tables

1 **Characteristics of the datasets used for internal validation, external validation and health association analyses** "Patient" indicates whether a cohort consists of sleep patients in a clinic. 26

2 **Hand-crafted features** 29

3 **Model performance metric definitions (TP: true positive; TN: true negative; FP: false positive; FN: false negative)** 30

4 **Sleep parameter definitions: total sleep duration (TSD), rapid-eye-movement (REM), non-rapid-eye-movement (NREM), sleep onset latency (SOL), wake after sleep onset (WASO), and sleep efficiency (SE).** 31

5 **Code table for UK Biobank variables used in the study.** 32

6 **Subject-wise sleep stage classification for benchmark models using internal validation datasets with the Raine Study and the Newcastle cohort:** The random forest model was trained using hand-crafted features. SleepNet is the deep recurrent network without pre-training. SleepNet-SSL is the network pre-trained using self-supervision. Five-fold subject-wise performance metrics (mean ± SD) are reported using the internal validation data. REM: rapid-eye-movement sleep, NREM: non-rapid-eye-movement sleep, Kappa score: κ. 36

8 **Model characteristics on the internal validation datasets (wake versus sleep):** subject-wise performance metrics (mean ± SD) are reported using the internal validation data. Sen: sensitivity, Spe: specificity. Wake is the negative class and the sleep is the positive class when calculating model performance. 38

9 **Model characteristics on the internal validation datasets (wake versus REM versus NREM):** subject-wise performance metrics (mean ± SD) are reported using the internal validation data. REM: rapid-eye-movement, NREM: non-rapid-eye-movement, Kappa score: κ. 39

10 **Model characteristics on the internal validation datasets (wake versus REM versus NREM I, II, III):** subject-wise performance metrics (mean ± SD) are reported using the internal validation data. REM: rapid-eye-movement, NREM: non-rapid-eye-movement, Kappa score: κ. 40

List of Figures

1 **Sleep stage distribution for all the datasets used.** 27

medRxiv preprint doi: https://doi.org/10.1101/2023.07.07.23292251; this version posted July 8, 2023. The copyright holder for this preprint (which was not certified by peer review) is the author/funder, who has granted medRxiv a license to display the preprint in perpetuity.
It is made available under a CC-BY 4.0 International license .

2 **How the intraclass correlation coefficient (ICC) changes with respect to the non-wear hours (h) (left) and the number of wear days (right) in a reliability simulation using data from 27,870 participants that had zero non-wear time across a seven-day period.** Mean and 95% confidence intervals are plotted. 34

3 **The distribution of non-wear time for all the participants from the UK Biobank.** 35

4 **Receiver operating characteristics curves for two-class (wake/sleep) and three-class (wake/REM/NREM) settings on the internal validation dataset using our best performing model self-supervised SleepNet.** REM: rapid-eye-movement sleep, NREM: non-rapid-eye-movement sleep. 37

5 **Agreement assessment via Bland-Altman plots for internal validation: total sleep duration (TSD), non-rapid-eye-movement sleep (NREM), and rapid-eye-movement sleep (REM).** 42

6 **Agreement assessment via Bland-Altman plots for external validation: total sleep duration, wake after sleep onset (WASO), non-rapid-eye-movement sleep (NREM), and rapid-eye-movement sleep (REM).** 43

7 **Agreement assessment via Bland-Altman plots for internal validation: non-rapid-eye-movement sleep (NREM) ratio, and rapid-eye-movement sleep (REM) ratio.** 44

8 **Agreement assessment via Bland-Altman plots for external validation: non-rapid-eye-movement sleep (NREM) ratio, and rapid-eye-movement sleep (REM) ratio.** 45

9 **Agreement assessment via Bland-Altman plots for internal validation: wake after sleep onset (WASO), and sleep efficiency (SE).** 46

10 **Agreement assessment via Bland-Altman plots for internal validation: wake after sleep onset (WASO), and sleep efficiency (SE).** 47

11 **Three class classification (wake/REM/NREM) confusion matrix**: epoch-to-epoch Kappa and balanced accuracies are shown. The number of predictions and proportion ratios are shown for each pair of ground-truth and prediction class. REM: rapid-eye-movement sleep; NREM: non-rapid-eye-movement sleep. 48

12 **Three-class sleep staging (wake/REM/NREM) for internal validation: epoch-to-epoch Kappa and balanced accuracies are shown.** The number of predictions and proportion ratios are shown for each pair of ground-truth and prediction class. REM: rapid-eye-movement sleep; NREM: non-rapid-eye-movement sleep. 49

medRxiv preprint doi: https://doi.org/10.1101/2023.07.07.23292251; this version posted July 8, 2023. The copyright holder for this preprint (which was not certified by peer review) is the author/funder, who has granted medRxiv a license to display the preprint in perpetuity.
It is made available under a CC-BY 4.0 International license .

13 **Five-class sleep staging (wake/REM/N1/N2/N3) for internal validation: epoch-to-epoch kappa and balanced accuracies are shown.** The number of predictions and proportion ratios are shown for each pair of ground-truth and prediction class. REM: rapid-eye-movement sleep, N1, N2, N3: non-rapid-eye-movement sleep 1, 2, 3. 50

14 **Three-class sleep staging (wake/REM/NREM) for external validation: epoch-to-epoch kappa and balanced accuracies are shown.** The number of predictions and proportion ratios are shown for each pair of ground-truth and prediction class. REM: rapid-eye-movement sleep; NREM: non-rapid-eye-movement sleep. 51

15 **Five-class sleep staging (wake/REM/N1/N2/N3) for external validation: epoch-to-epoch kappa and balanced accuracies are shown.** The number of predictions and proportion ratios are shown for each pair of ground-truth and prediction class. REM: rapid-eye-movement sleep, N1, N2, N3: non-rapid-eye-movement sleep 1, 2, 3. 51

16 **A sample actigram, hypnogram ground truth and prediction for a participant whose sleep stages are well captured:** the **top** hypnogram is the ground-truth and the **bottom** hypnogram is the prediction generated by SleepNet based on the actigram. REM: rapid-eye-movement sleep, N1, N2, N3: non-rapid-eye-movement sleep 1, 2, 3. 52

17 **Participant flow diagram for the analysis of sleep and all-cause mortality in the UK Biobank.** TDI: Townsend deprivation index, BMI: body mass index, SR_health: self-reported overall health, SR_insomnia: self-reported insomnia symptoms, CVD: Cardiovascular disease. 54

18 **Correlation matrix for device-measured and self-reported sleep parameters on the UK Biobank.** The self-reported total sleep duration was obtained via questionnaire at baseline assessment in the UK Biobank. REM: rapid-eye-movement sleep, NREM: non-rapid-eye-movement sleep. 55

19 **Box plots showing the distributions of device-measured overnight sleep duration against self-reported total sleep duration.** The box whiskers reflect the lowest and highest data points that are 1.5 times of the inter-quartile-range from the median. 56

21 **Adjusted marginal mean (95% confidence interval) device-measured mean overnight sleep duration and mean sleep efficiency by self-reported overall health status and insomnia history in the UK Biobank.** Mean overnight sleep duration and sleep efficiency were adjusted for age and sex. 57

medRxiv preprint doi: https://doi.org/10.1101/2023.07.07.23292251; this version posted July 8, 2023. The copyright holder for this preprint (which was not certified by peer review) is the author/funder, who has granted medRxiv a license to display the preprint in perpetuity.
It is made available under a CC-BY 4.0 International license .

22 **Device-measured sleep probability trajectories throughout the day for the UK Biobank participants (weekday vs weekend).** Top: variations of the average overnight sleep probability for the participants with self-reported "morning" and "evening" chronotype (a) and the overnight sleep distributions across thirds of device-measured physical activity level (b). Bottom: variations of the average REM (c) and NREM (d) probability in participants with a history of self-reported insomnia symptoms versus those without. Rapid-eye-movement sleep (REM), and non-rapid-eye-movement sleep (NREM). Areas of squares represent the inverse of the variance of the log risk. And the I bars denote the 95% confidence interval for the floated risks. 58

24 **Associations of overnight sleep duration with all-cause mortality for groups with low and high sleep efficiency additionally adjusted for body mass index.** The model used 1,642 events among 62,214 participants. We used age as the timescale and adjusted for sex, ethnicity, Townsend Deprivation Index of baseline address (split by quarter in the study population), educational qualifications, smoking status, alcohol consumption (Never, <3 times/week, 3+ times/week), overall activity (measured in milli-gravity units). Areas of squares represent the inverse of the variance of the log risk. The I bars denote the 95% confidence interval for the floated risks. 60

25 **Associations of overnight sleep duration (a) and sleep efficiency (b) with all-cause mortality additionally adjusted for body mass index.** The model used 1,642 events among 62,214 participants. We used age as the timescale and adjusted for sex, ethnicity, Townsend Deprivation Index of baseline address (split by quarter in the study population), educational qualifications, smoking status, alcohol consumption (Never, <3 times/week, 3+ times/week), overall activity (measured in milli-gravity units), and body mass index. Areas of squares represent the inverse of the variance of the log risk. The I bars denote the 95% confidence interval for the floated risks. 61

26 **Associations of device-measured overnight sleep duration and all-cause mortality with greater granularity.** The model used 1,642 events among 62,214 participants. We used age as the timescale and adjusted for sex, ethnicity, Townsend Deprivation Index of baseline address (split by quarter in the study population), educational qualifications, smoking status, alcohol consumption (Never, <3 times/week, 3+ times/week), and overall activity (measured in milli-gravity units). Areas of squares represent the inverse of the variance of the log risk. The I bars denote the 95% confidence interval for the floated risks. 62

medRxiv preprint doi: https://doi.org/10.1101/2023.07.07.23292251; this version posted July 8, 2023. The copyright holder for this preprint (which was not certified by peer review) is the author/funder, who has granted medRxiv a license to display the preprint in perpetuity.
It is made available under a CC-BY 4.0 International license .

Table 1: **Characteristics of the datasets used for internal validation, external validation and health association analyses** "Patient" indicates whether a cohort consists of sleep patients in a clinic.

Name	n	Age	Placement	Device	Patient	Publication
UK Biobank	103,561	62.3 ± 7.9	Dom wrist	Axivity	✗	[1]
Raine Gen1	865	56.7 ± 5.6	Dom wrist	GT3X	✗	[2]
Raine Gen2	795	22.1 ± 0.6	Dom wrist	GT3X	✗	[2]
Newcastle	28	44.9 ± 14.9	Both wrists	GENEActiv	✓	[3]
Leicester	30	30.8 ± 6.7	Both wrists	Axivity	✗	[4]
Pennsylvania	22	22.8 ± 4.5	Non-dom wrist	Axivity	✗	[5]

5. Datasets

Raine Study. The Raine Study has followed up roughly 2900 children since 1989 in Australia. A subset of children (Raine Gen2, 50% females) at the age of 22 and their parents (Raine Gen1, 57% females) were invited to undergo one night of laboratory-based polysomnography at Western Australia's Center for Sleep Science [2, 6]. Every participant was instructed to wear an ActiGraph GT3X device on the dominant wrist. Earlier GT3X firmware would enter an idle mode to save the battery when no sufficient movement was detected, so we only included participants with no missing data for the Raine Gen2 cohort.

Newcastle. The Newcastle dataset recruited 28 adult patients (39% females) for a one night laboratory-based polysomnography assessment in Newcastle upon Tyne, UK, as part of their routine clinical visit [3]. During the polysomnography recording, the participants wore two GENEActive devices, one on each wrist. The sampling frequency for the wristbands was set to 85.7 Hz.

Leicester. Thirty healthy volunteers (63% females and 73% white) wore three devices: GENEActive, Axivity AX3, and ActiGraph GT9X on each wrist during one night of laboratory-based polysomnography assessment [4]. The relative position of the devices was randomly allocated for each participant. The devices were set to record at 100 Hz. During the lab visit, when the participants wished to go to bed, the recording was started. The sleep episodes usually ended between 6 am and 7

medRxiv preprint doi: https://doi.org/10.1101/2023.07.07.23292251; this version posted July 8, 2023. The copyright holder for this preprint (which was not certified by peer review) is the author/funder, who has granted medRxiv a license to display the preprint in perpetuity.
It is made available under a CC-BY 4.0 International license .

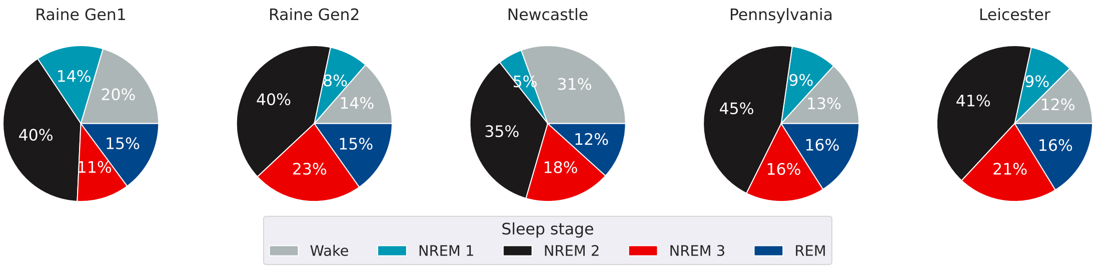

Figure 1: **Sleep stage distribution for all the datasets used.**

am the following morning. We cleaned up the recording sessions such that every recording would start from ”light off” and end at ”light off” to ensure comparability.

Pennsylvania. The Pennsylvania dataset consists of 22 healthy sleepers who had one-night of laboratory-based polysomnography assessment at the University of Pennsylvania Center for sleep [5]. The participants were asked to wear an Axivity device on the non-dominant wrist during the polysomnography session.

UK Biobank. The UK Biobank is a longitudinal cohort study that recruited 500,000 adults from the UK [7]. A subset of the participants was invited to wear an Axivity device on the dominant wrist for one week in a free-living environment [1]. The sampling rate was set to 100 Hz. Roughly 100,000 participants (56% females) consented and participated in the accelerometry study. Other than the accelerometry data, a rich set of biomedical information was also collected on the study participants, such as health record linkage, self-reported questionnaire and genetic data.

We preprocessed all the datasets by manual quality checks for unrealistic high values for accelerometry (>200 mg), parsing successes, polysomnography alignment, and visual inspection.

6. Model development

6.1. Self-supervised pre-training

To obtain a feature extractor by leveraging a large amount of unlabelled data from the UK Biobank, we applied multi-task self-supervised learning following [8]. In self-supervision pre-training, the model was designed to discriminate whether a

medRxiv preprint doi: https://doi.org/10.1101/2023.07.07.23292251; this version posted July 8, 2023. The copyright holder for this preprint (which was not certified by peer review) is the author/funder, who has granted medRxiv a license to display the preprint in perpetuity.
It is made available under a CC-BY 4.0 International license .

set of binary transformations have been applied to the signal. We selected reversal, permutation, and time-warping as potential self-supervised learning because they are suitable for learning spatiotemporal patterns.

The feature extractor was built on top of ResNet-17 V2 [9] with 1D convolution, in total, with 10M parameters. Each feature vector is of size 1024. We used cross-entropy as the cost function, with each task having the same weight to balance the features learned from each task. In the training procedure, we applied axis swap and rotation as data augmentation to obtain a representation that is orientation invariant. During training time, we used a batch size of 2000 as a larger batch size was found to produce features with better quality. Adam [10] was used for optimisation with a learning rate of 1e-3. We distributed the training across 4 Tesla V100-SXM2 GPUs with 32GB. Early-stopping with a patience of five steps was used to avoid overfitting. It took about 420 GPU hours for the model to converge. More details can be found in [8].

6.2. SleepNet training

We used the pre-trained ResNet from self-supervision as the base model for feature extraction. Then, we appended two layers of Bi-directional Long-Short-Term-Memory (LSTM) layers of 1024 units to learn the temporal dependencies of the model [11]. In the end, we had two fully-connected layers of 512 units to generate the sleep stages. The model was trained to discriminate five sleep stages directly (wake, N1, N2, N3 and REM). To obtain the three-class output, we combined NREM I, II, and III into the NREM class. Likewise, we combined NREM I, II, III and NREM into the sleep class to obtain the two-class output.

The learning rate was set to be 1e-3. We also set the gradient clapping to 1 to avoid exploding gradient for LSTM. We used weighted Cross-Entropy as the objective function and weighted each class with the inverse of its frequency to account for the imbalanced dataset. We also used rotation and axis swap to augment the input data to obtain a direction-invariant model. Each training mini-batch consisted of five participants. For each individual, we selected four 1.5-hour sequences with random starting points to avoid overfitting to the study protocol, where the beginning and the end of the sequence are always the “wake” class. The model was trained on a

medRxiv preprint doi: https://doi.org/10.1101/2023.07.07.23292251; this version posted July 8, 2023. The copyright holder for this preprint (which was not certified by peer review) is the author/funder, who has granted medRxiv a license to display the preprint in perpetuity.
It is made available under a CC-BY 4.0 International license .

Tesla V100-SXM2 with 32GB of memory. It took about 12 hours for the model to converge. The model performance was reported using five-fold subject-wise cross-validation. We first split the data into train/test with a ratio of 8:2. We further split the train set into train/validation with a ratio of 8:2. We used early stopping with a patience of ten steps to avoid overfitting on the validation set in each cross-validation fold.

Table 2: **Hand-crafted features**

Handcrafted features	Notes
Sleep features [12]	
	All sleep features have 12 derived variables:
ENMO	mean, std, min, max, entropy 20 bins (low resolution),
Angle Z	entropy 200 bins (high resolution), median absolute derivation,
Locomotor inactivity during sleep	and mean difference between neighbouring windows.
Axis features [13]	
Mean	1 per axis
Standard deviation	1 per axis
Range	1 per axis
Inter-quantile-range	1 per axis
Correlation of variations	1 per axis
Features on the vector norm [13]	norm $= \sqrt{x^2 + y^2 + z^2}$
Mean	
Standard deviation	
Inter-quantile-range	
Median absolute derivation	
Kurtosis	
Skew	
Truncated ENMO	
Absolute value of ENMO	
Entropy	
Dominant Frequency	
Total power	
Dominant frequencies	3 features: 0.3-5 Hz, 0.3-15 Hz, and 0.6-2.5 Hz
Dominant frequency power	3 features: 0.3-5 Hz, 0.3-15 Hz, and 0.6-2.5 Hz
Second dominant frequency	1 feature: 0.3-15 Hz
Fourier transform coefficients	11 features: 1 Hz - 11 Hz
Fourier coefficients	12 features: 1st - 12th coefficient

medRxiv preprint doi: https://doi.org/10.1101/2023.07.07.23292251; this version posted July 8, 2023. The copyright holder for this preprint (which was not certified by peer review) is the author/funder, who has granted medRxiv a license to display the preprint in perpetuity.
It is made available under a CC-BY 4.0 International license .

Table 3: **Model performance metric definitions (TP: true positive; TN: true negative; FP: false positive; FN: false negative)**

Metric	Definition
Precision	$\frac{\text{TP}}{\text{TP+FP}}$
Sensitivity/Recall	$\frac{\text{TP}}{\text{TP+FN}}$
Specificity	$\frac{\text{TN}}{\text{TN+FP}}$
Accuracy	$\frac{\text{TP+TN}}{\text{TP+TN+FP+FN}}$
F1	$2 \times \frac{\text{precision}\cdot\text{recall}}{\text{precision+recall}}$
Kappa	$1 - \frac{1-p_o}{1-p_e}$<br>p_o: relative observed agreement<br>p_e: expected agreement probability
Balanced accuracy	$\frac{1}{n}\sum_i \text{Accuracy}_{class_i}$

medRxiv preprint doi: https://doi.org/10.1101/2023.07.07.23292251; this version posted July 8, 2023. The copyright holder for this preprint (which was not certified by peer review) is the author/funder, who has granted medRxiv a license to display the preprint in perpetuity.
It is made available under a CC-BY 4.0 International license .

Table 4: **Sleep parameter definitions: total sleep duration (TSD), rapid-eye-movement (REM), non-rapid-eye-movement (NREM), sleep onset latency (SOL), wake after sleep onset (WASO), and sleep efficiency (SE).**

Parameter	Definition
Total sleep duration (TSD)	The total time spent in sleep during the recording period per day.
Overnight sleep duration	The longest sleep window duration (max one hour of sleep discontinuity allowed) over a noon-to-noon interval.
Time in bed	The amount of time spent in bed: A person might not be asleep during this period. Our time in bed was estimated using a random forest model that was trained using data from sleep diaries.
Sleep onset latency (SOL)	The time difference between when one gets in bed and the sleep onset. The sleep onset (SOL) is defined as the first occurrence of three consecutive 30-sec sleep windows.
Wake after sleep onset (WASO)	The amount of wake time spent after the sleep onset during the longest sleep window.
Sleep efficiency (SE)	SE for sleep window after device-detected sleep onset: $\frac{\text{Overnight sleep duration}}{\text{time in bed}}$
REM duration	The total time spent in the REM stage.
REM ratio	$\frac{\text{REM duration}}{\text{TSD}}$
NREM duration	The total time spent in the NREM I, II, and III stages.
NREM ratio	$\frac{\text{NREM duration}}{\text{TSD}}$

medRxiv preprint doi: https://doi.org/10.1101/2023.07.07.23292251; this version posted July 8, 2023. The copyright holder for this preprint (which was not certified by peer review) is the author/funder, who has granted medRxiv a license to display the preprint in perpetuity.
It is made available under a CC-BY 4.0 International license .

7. UK Biobank analysis

Table 5: **Code table for UK Biobank variables used in the study.**

Variable	Code name
Month of birth	p52
Year of birth	p34
Device wear time	p90010
Sex	p31
Ethnicity	p21000
Smoking status	p20116
Alcohol consumption	p1558
Education qualification	p6138
Body mass index	p21001
Employment status	p6142
Overall health rating	p2178
Self-reported total sleep duration	p1160
Townsend Deprivation Index	p189
Overall accelerometry average	p90012
Self-reported trouble falling/ staying asleep	p1200

The UK Biobank variable codes are shown in Table 5. We used the month of birth (p52) and year of birth (p34) along with device wear time (p90010) to compute the age at wear time. Participants were asked about their insomnia symptoms history (p1200) by "Do you have trouble falling asleep at night or do you wake up in the middle of the night?". Four responses were possible: "never/rarely", "sometimes", "usually", and "prefer not to answer".

7.1. Sleep and all-cause mortality

The relationship between machine learning-derived sleep architecture estimates and all-cause mortality was assessed using association analyses. The main analysis split the participants into six groups stratified by sleep efficiency cut-off with clinical relevance. Then, five groups were created based on exact hour cut-offs in line with sleep recommendation guidelines for overnight sleep duration [14]. Four groups were created based on percentage cut-offs of clinical relevance for sleep efficiency [15]. In

medRxiv preprint doi: https://doi.org/10.1101/2023.07.07.23292251; this version posted July 8, 2023. The copyright holder for this preprint (which was not certified by peer review) is the author/funder, who has granted medRxiv a license to display the preprint in perpetuity.
It is made available under a CC-BY 4.0 International license .

the sensitivity analysis, seven sleep groups were created on exact hour cut-offs to capture the variations in participants with lower and higher sleep durations.

Mortality was determined using death registry data (obtained by UK Biobank from NHS Digital for participants in England and Wales and from the NHS Central Register, National Records of Scotland, for participants in Scotland). For survival analyses, participants were censored at the earliest of UK Biobank's record censoring date for mortality data (2021-09-30 for participants in England and Wales and 2021-10-31 for participants in Scotland, with country assigned based on baseline assessment centre) and a record of loss to linked health record follow-up (field 191; 2 participants only).

In addition to the exclusions described for the analyses above, for prospective analyses for incident mortality we further excluded the participants if they had a prior hospitalisation for restless syndrome, any cardiovascular disease or cancer (a hospital episode with primary diagnosis G473, I00-I99 or C00-C99).

Models used age as the timescale, and the main analysis was adjusted for sex (male/female), ethnicity (white/non-white), Townsend Deprivation Index of baseline address (split by quarter in the study population), educational qualifications (school leaver, further education, higher education), smoking status (never smoker, ex-smoker, current smoker), alcohol consumption (never, <3 times/week, 3+ times/week), and overall activity (measured in milli-gravity units). An additional analysis further adjusted for BMI (categorised as <18.5 kg/m2, 18.5-24.9 kg/m2, 25.0-29.9 kg/m2, 30+ kg/m2). See Supplementary Table 5 for UK Biobank fields).

Results are presented with their 95% confidence intervals. The Floating Absolute Risk approach was used to calculate confidence intervals for the estimate in each group, without contrast to a reference group [16, 17, 18].

In statistical testing using the Grambsch-Therneau test with the Kaplan-Meier transformation, there was some evidence that the joint associations of overnight sleep duration and sleep efficiency with incident mortality violated the proportional hazards assumption (with age as the timescale). However, assessing associations at younger (< 65 years) and older (≥ 65 years) ages did not suggest substantially differing associations by age, and so the overall hazard ratios are presented.

medRxiv preprint doi: https://doi.org/10.1101/2023.07.07.23292251; this version posted July 8, 2023. The copyright holder for this preprint (which was not certified by peer review) is the author/funder, who has granted medRxiv a license to display the preprint in perpetuity.
It is made available under a CC-BY 4.0 International license .

7.2. Reliability assessment for device wear time exclusion criterion

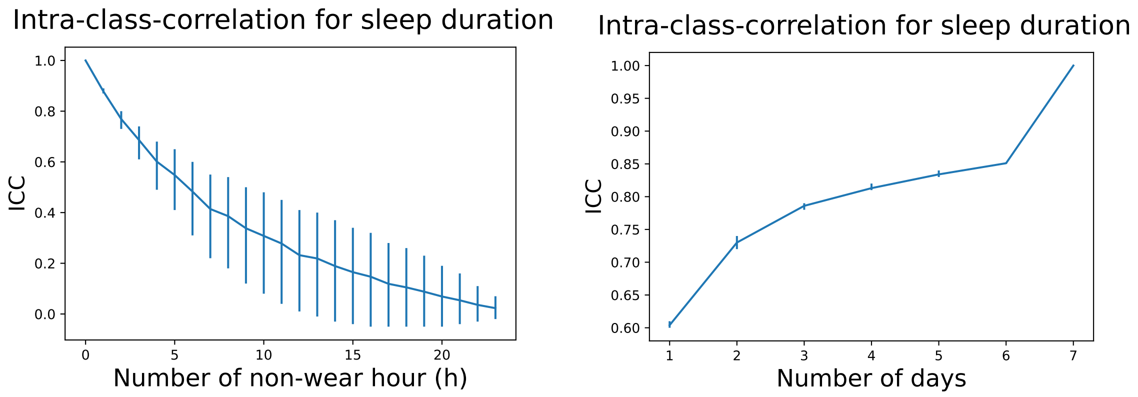

Figure 2: **How the intraclass correlation coefficient (ICC) changes with respect to the non-wear hours (h) (left) and the number of wear days (right) in a reliability simulation using data from 27,870 participants that had zero non-wear time across a seven-day period.** Mean and 95% confidence intervals are plotted.

We needed to discard participants with too much non-wear time to obtain a stable sleep duration estimate. Ideally, all the participants would have perfect seven-day device wear, which was not the case. Thus, we needed to determine the minimum wear time for seven days so that there is a high agreement between sleep duration computed for participants with perfect data and those computed for participants with missing data. To do this, we first selected a subset of 27,870 participants who did not have any non-wear time during the seven-day window. Then, we simulated the missing data by randomly removing one hour from each day or one whole day of data from each week from their recordings. We increased the amount of simulated missing data step-wise until all the data was removed. Then, we compared weekly mean sleep durations computed on data before and after removing the simulated missing periods.

We used the intraclass correlation coefficient (ICC) to determine the acceptable missing time threshold. We selected two-way random-effects, single rater with an absolute agreement, ICC2, to reflect the reliability of our sleep duration measurement if we have missing data in the measurements [19]. Supplementary Figure 2 depicts

medRxiv preprint doi: https://doi.org/10.1101/2023.07.07.23292251; this version posted July 8, 2023. The copyright holder for this preprint (which was not certified by peer review) is the author/funder, who has granted medRxiv a license to display the preprint in perpetuity.
It is made available under a CC-BY 4.0 International license .

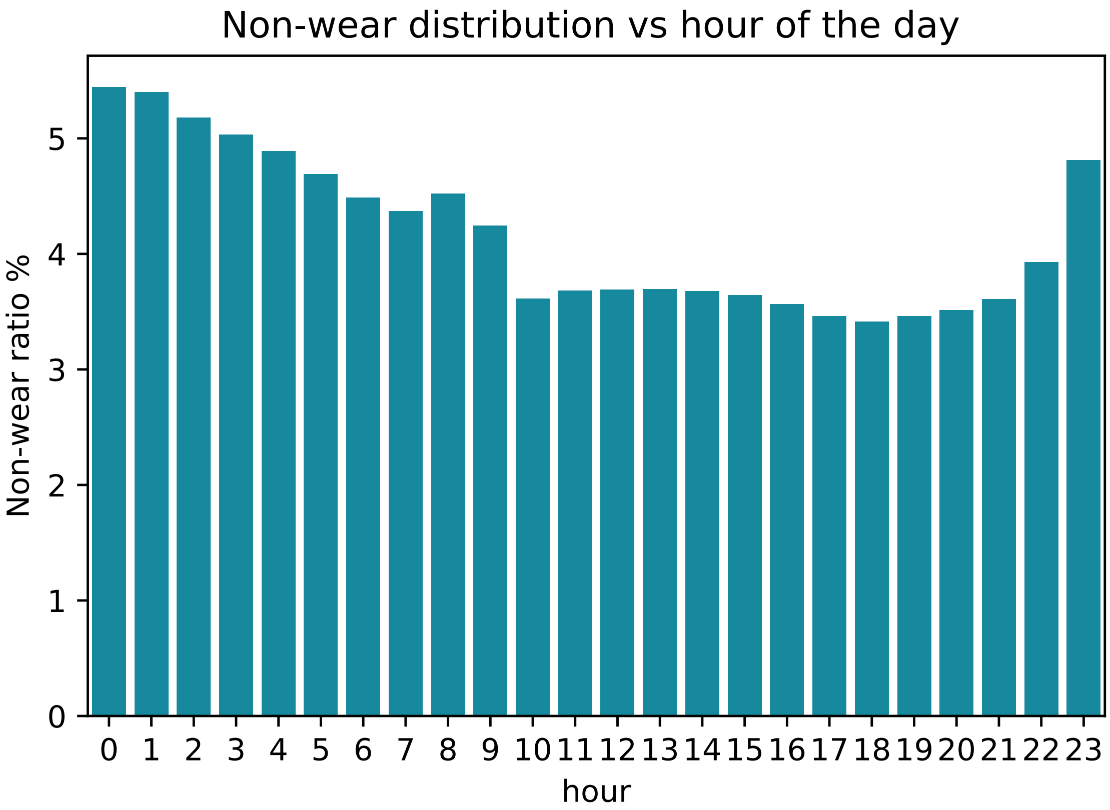

Figure 3: **The distribution of non-wear time for all the participants from the UK Biobank.**

the ICC mean and 95% confidence intervals for the missing non-wear hour (Supplementary Figure 2 Left)and missing days (Supplementary Figure 2 Right). We used an ICC of 0.75 threshold when deciding the acceptable device wear range. According to the 0.75 cut-off, a maximum of two non-wear hours per day and a minimum of three days per week are suitable for obtaining stable measurements of sleep duration.

medRxiv preprint doi: https://doi.org/10.1101/2023.07.07.23292251; this version posted July 8, 2023. The copyright holder for this preprint (which was not certified by peer review) is the author/funder, who has granted medRxiv a license to display the preprint in perpetuity. It is made available under a CC-BY 4.0 International license .

8. Additional Results

8.1. Model performance

Table 6: **Subject-wise sleep stage classification for benchmark models using internal validation datasets with the Raine Study and the Newcastle cohort:** The random forest model was trained using hand-crafted features. SleepNet is the deep recurrent network without pre-training. SleepNet-SSL is the network pre-trained using self-supervision. Five-fold subject-wise performance metrics (mean ± SD) are reported using the internal validation data. REM: rapid-eye-movement sleep, NREM: non-rapid-eye-movement sleep, Kappa score: κ.

Model	Sleep versus Wake			Wake versus REM versus NREM		
	κ	Accuracy	F1	κ	Accuracy	F1
Random forest [13, 12]	0.472±0.192	0.756±0.102	0.729±0.104	0.290±0.149	0.507±0.072	0.464±0.072
SleepNet	0.468±0.193	0.757±0.103	0.727±0.105	0.313±0.162	0.576±0.112	0.535±0.106
SleepNet-SSL	**0.511±0.196**	**0.775±0.105**	**0.750±0.107**	**0.375±0.163**	**0.625±0.116**	**0.573±0.116**

Supplementary Table 6 shows the model performance comparison between the random forest model that used hand-crafted features and our proposed SleepNet on the internal validation. SleepNet pre-trained with self-supervision had the best performance in both the two-class ($\kappa = 0.511 \pm 0.196$) and three-class settings ($\kappa = 0.375 \pm 0.163$). In addition, the area under the receiver operating characteristic curve for the best SleepNet model is0.88 for the two-class setting and0.81 for the three-class setting (Supplementary Figure 4).

medRxiv preprint doi: https://doi.org/10.1101/2023.07.07.23292251; this version posted July 8, 2023. The copyright holder for this preprint (which was not certified by peer review) is the author/funder, who has granted medRxiv a license to display the preprint in perpetuity.
It is made available under a CC-BY 4.0 International license .

Table 7: **Subject-wise performance sleep classification validation using our best-performing model:** All the performance is reported within period in bed. Cohort-specific and pooled performance (Kappa (κ), balanced accuracy, and F1) are shown for both internal and external validation. The pooled performance is calculated by combining all the participants from different datasets. REM: rapid-eye-movement sleep; NREM: non-rapid-eye-movement sleep.

Dataset	Sleep versus Wake			Wake versus REM versus NREM		
	κ	Accuracy	F1	κ	Accuracy	F1
Internal validation						
Raine Gen1	0.561±0.161	0.791±0.091	0.775±0.089	0.389±0.152	0.623±0.108	0.586±0.105
Raine Gen2	0.437±0.189	0.758±0.101	0.712±0.100	0.344±0.161	0.603±0.115	0.552±0.108
Newcastle	0.394±0.189	0.715±0.091	0.686±0.103	0.285±0.151	0.513±0.078	0.467±0.085
**Pooled internal**	0.509±0.184	0.777±0.097	0.748±0.099	0.369±0.158	0.613±0.112	0.571±0.108
External Validation						
Leicester	0.278±0.141	0.678±0.072	0.633±0.075	0.253±0.122	0.527±0.086	0.488±0.082
Pennsylvania	0.468±0.225	0.807±0.117	0.725±0.118	0.374±0.172	0.626±0.092	0.565±0.097
**Pooled external**	0.360±0.205	0.734±0.114	0.673±0.106	0.306±0.157	0.570±0.101	0.521±0.097

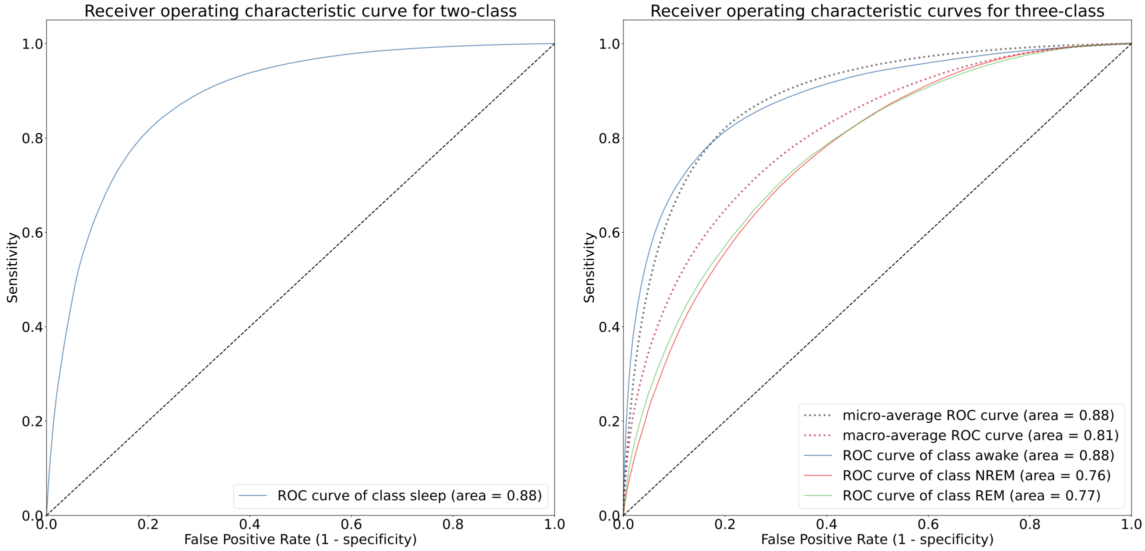

Figure 4: **Receiver operating characteristics curves for two-class (wake/sleep) and three-class (wake/REM/NREM) settings on the internal validation dataset using our best performing model self-supervised SleepNet.** REM: rapid-eye-movement sleep, NREM: non-rapid-eye-movement sleep.

medRxiv preprint doi: https://doi.org/10.1101/2023.07.07.23292251; this version posted July 8, 2023. The copyright holder for this preprint (which was not certified by peer review) is the author/funder, who has granted medRxiv a license to display the preprint in perpetuity.
It is made available under a CC-BY 4.0 International license .

Table 8: **Model characteristics on the internal validation datasets (wake versus sleep):** subject-wise performance metrics (mean ± SD) are reported using the internal validation data. Sen: sensitivity, Spe: specificity. Wake is the negative class and the sleep is the positive class when calculating model performance.

Wake versus Sleep									
Subgroups	Raine Gen1			Raine Gen2			Newcastle		
	n	Sen (%)	Spe (%)	n	Sen (%)	Spe (%)	n	Sen (%)	Spe (%)
Sex									
Male	357	92.0 ± 9.1	63.7 ± 20.4	264	87.6 ± 10.0	62.7 ± 21.8	15	79.6 ± 21.9	59.1 ± 24.9
Femal	459	91.5 ± 9.2	68.6 ± 21.3	273	88.8 ± 9.3	63.3 ± 22.3	7	82.9 ± 14.9	69.3 ± 14.0
Body Mass Index (BMI)									
< 25	232	92.9 ± 8.5	63.7 ± 22.1	338	88.5 ± 9.3	62.8 ± 21.6	-	-	-
25 - 29.9	318	92.1 ± 8.8	65.8 ± 21.0	120	89.7 ± 8.7	62.8 ± 22.7	-	-	-
>30	265	90.1 ± 9.9	69.7 ± 19.7	79	84.9 ± 11.8	64.0 ± 23.1	-	-	-
Apnea Hypopnea Index (AHI)									
< 5	199	93.7 ± 7.2	67.0 ± 20.7	338	88.0 ± 10.0	65.2 ± 21.6	-	-	-
5 - 14.9	349	91.5 ± 8.4	67.6 ± 21.2	146	88.9 ± 9.4	58.4 ± 23.0	-	-	-
15 - 29.9	150	90.9 ± 9.3	66.5 ± 20.8	39	88.5 ± 8.1	61.7 ± 20.5	-	-	-
≥ 30	114	89.9 ± 12.2	62.5 ± 20.4	14	84.9 ± 8.8	62.4 ± 21.5	-	-	-
Has sleep disorder(s)?									
Yes	155	90.6 ± 10.1	64.5 ± 22.3	106	87.6 ± 10.0	65.2 ± 22.2	15	75.3 ± 21.5	66.0 ± 23.4
No	661	91.9 ± 8.9	66.9 ± 20.7	431	88.4 ± 9.6	62.5 ± 22.0	7	92.3 ± 6.0	54.4 ± 18.3

medRxiv preprint doi: https://doi.org/10.1101/2023.07.07.23292251; this version posted July 8, 2023. The copyright holder for this preprint (which was not certified by peer review) is the author/funder, who has granted medRxiv a license to display the preprint in perpetuity.
It is made available under a CC-BY 4.0 International license .

Table 9: **Model characteristics on the internal validation datasets (wake versus REM versus NREM):** subject-wise performance metrics (mean ± SD) are reported using the internal validation data. REM: rapid-eye-movement, NREM: non-rapid-eye-movement, Kappa score: κ.

	Wake versus REM versus NREM					
Subgroups	Raine Gen1		Raine Gen2		Newcastle	
	n	κ	n	κ	n	κ
Sex						
Male	357	0.293 ± 0.100	264	0.286 ± 0.120	15	0.200 ± 0.137
Female	459	0.313 ± 0.114	273	0.284 ± 0.117	7	0.258 ± 0.084
Body Mass Index (BMI)						
< 25	232	0.375 ± 0.162	338	0.342 ± 0.148	-	-
25 - 29.9	318	0.390 ± 0.152	120	0.335 ± 0.170	-	-
>30	265	0.401 ± 0.143	79	0.329 ± 0.178	-	-
Apnea Hypopnea Index (AHI)						
< 5	199	0.397 ± 0.163	338	0.349 ± 0.156	-	-
5 - 14.9	349	0.390 ± 0.148	146	0.317 ± 0.158	-	-
15 - 29.9	150	0.395 ± 0.153	39	0.355 ± 0.166	-	-
≥ 30	114	0.369 ± 0.143	14	0.273 ± 0.139	-	-
Has sleep disorder(s)?						
Yes	155	0.386 ± 0.149	106	0.354 ± 0.162	15	0.277 ± 0.148
No	661	0.390 ± 0.153	431	0.335 ± 0.157	7	0.303 ± 0.179

medRxiv preprint doi: https://doi.org/10.1101/2023.07.07.23292251; this version posted July 8, 2023. The copyright holder for this preprint
(which was not certified by peer review) is the author/funder, who has granted medRxiv a license to display the preprint in perpetuity.
It is made available under a CC-BY 4.0 International license .

Table 10: **Model characteristics on the internal validation datasets (wake versus REM versus NREM I, II, III):** subject-wise performance metrics (mean ± SD) are reported using the internal validation data. REM: rapid-eye-movement, NREM: non-rapid-eye-movement, Kappa score: κ.

	Wake versus REM versus NREM I, II, III					
Subgroups	Raine Gen1		Raine Gen2		Newcastle	
	n	κ	n	κ	n	κ
Sex						
Male	357	0.279 ± 0.103	264	0.287 ± 0.120	16	0.014 ± 0.102
Female	459	0.307 ± 0.111	273	0.285 ± 0.113	9	0.125 ± 0.106
Body Mass Index (BMI)						
< 25	232	0.295 ± 0.117	338	0.286 ± 0.110	-	-
25 - 29.9	318	0.309 ± 0.107	120	0.292 ± 0.127	-	-
>30	265	0.307 ± 0.102	79	0.273 ± 0.140	-	-
Apnea Hypopnea Index (AHI)						
< 5	199	0.307 ± 0.114	338	0.293 ± 0.116	-	-
5 - 14.9	349	0.309 ± 0.108	146	0.264 ± 0.118	-	-
15 - 29.9	150	0.309 ± 0.104	39	0.299 ± 0.127	-	-
≥ 30	114	0.283 ± 0.104	14	0.274 ± 0.131	-	-
Has sleep disorder(s)?						
Yes	155	0.286 ± 0.107	106	0.297 ± 0.131	15	0.213 ± 0.136
No	661	0.309 ± 0.108	431	0.283 ± 0.115	7	0.230 ± 0.099

medRxiv preprint doi: https://doi.org/10.1101/2023.07.07.23292251; this version posted July 8, 2023. The copyright holder for this preprint (which was not certified by peer review) is the author/funder, who has granted medRxiv a license to display the preprint in perpetuity.
It is made available under a CC-BY 4.0 International license .

8.2. Cohort-specific performance against polysomnography using SleepNet

medRxiv preprint doi: https://doi.org/10.1101/2023.07.07.23292251; this version posted July 8, 2023. The copyright holder for this preprint (which was not certified by peer review) is the author/funder, who has granted medRxiv a license to display the preprint in perpetuity.
It is made available under a CC-BY 4.0 International license .

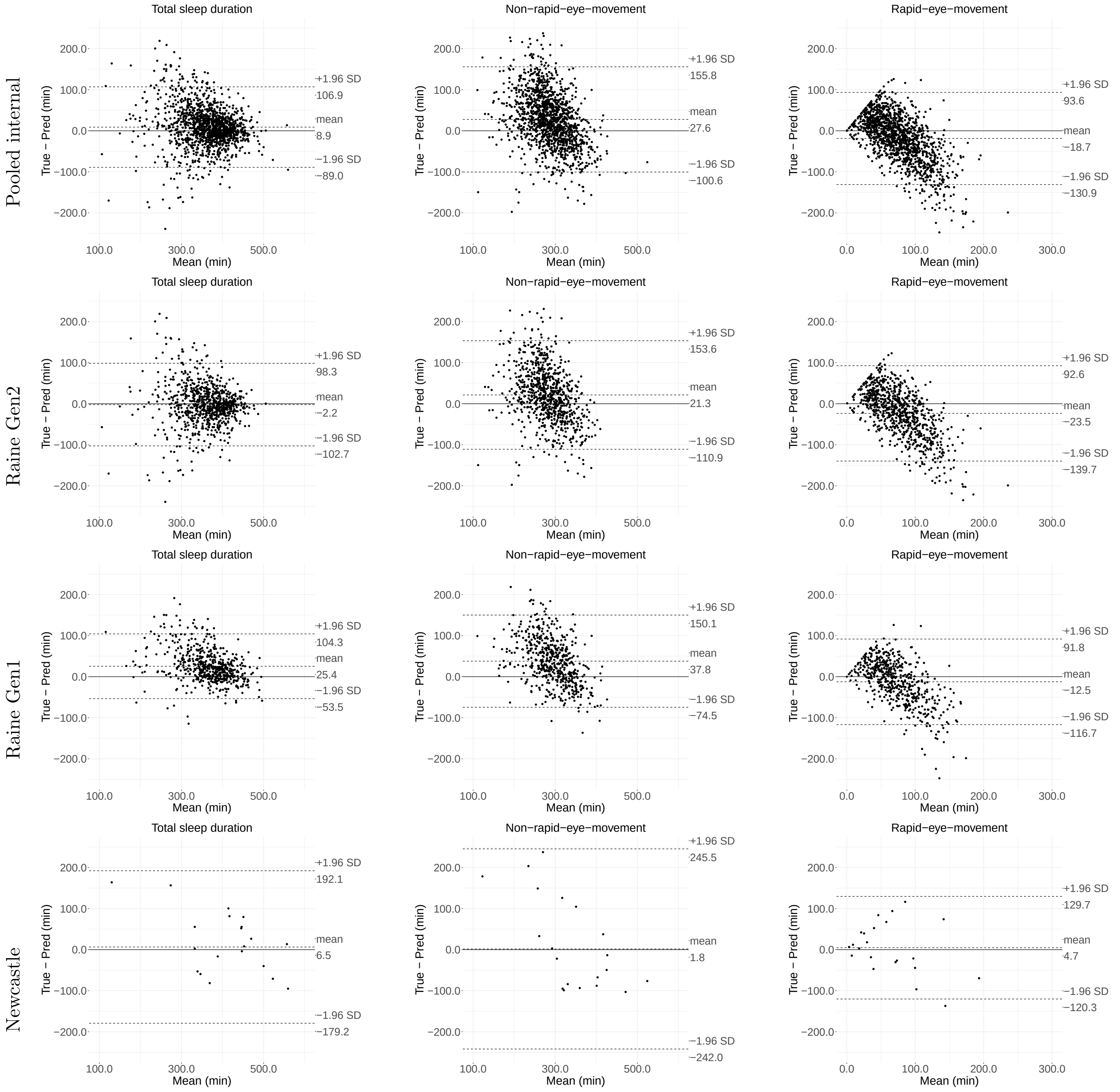

Figure 5: **Agreement assessment via Bland-Altman plots for internal validation: total sleep duration (TSD), non-rapid-eye-movement sleep (NREM), and rapid-eye-movement sleep (REM).**

medRxiv preprint doi: https://doi.org/10.1101/2023.07.07.23292251; this version posted July 8, 2023. The copyright holder for this preprint (which was not certified by peer review) is the author/funder, who has granted medRxiv a license to display the preprint in perpetuity.
It is made available under a CC-BY 4.0 International license .

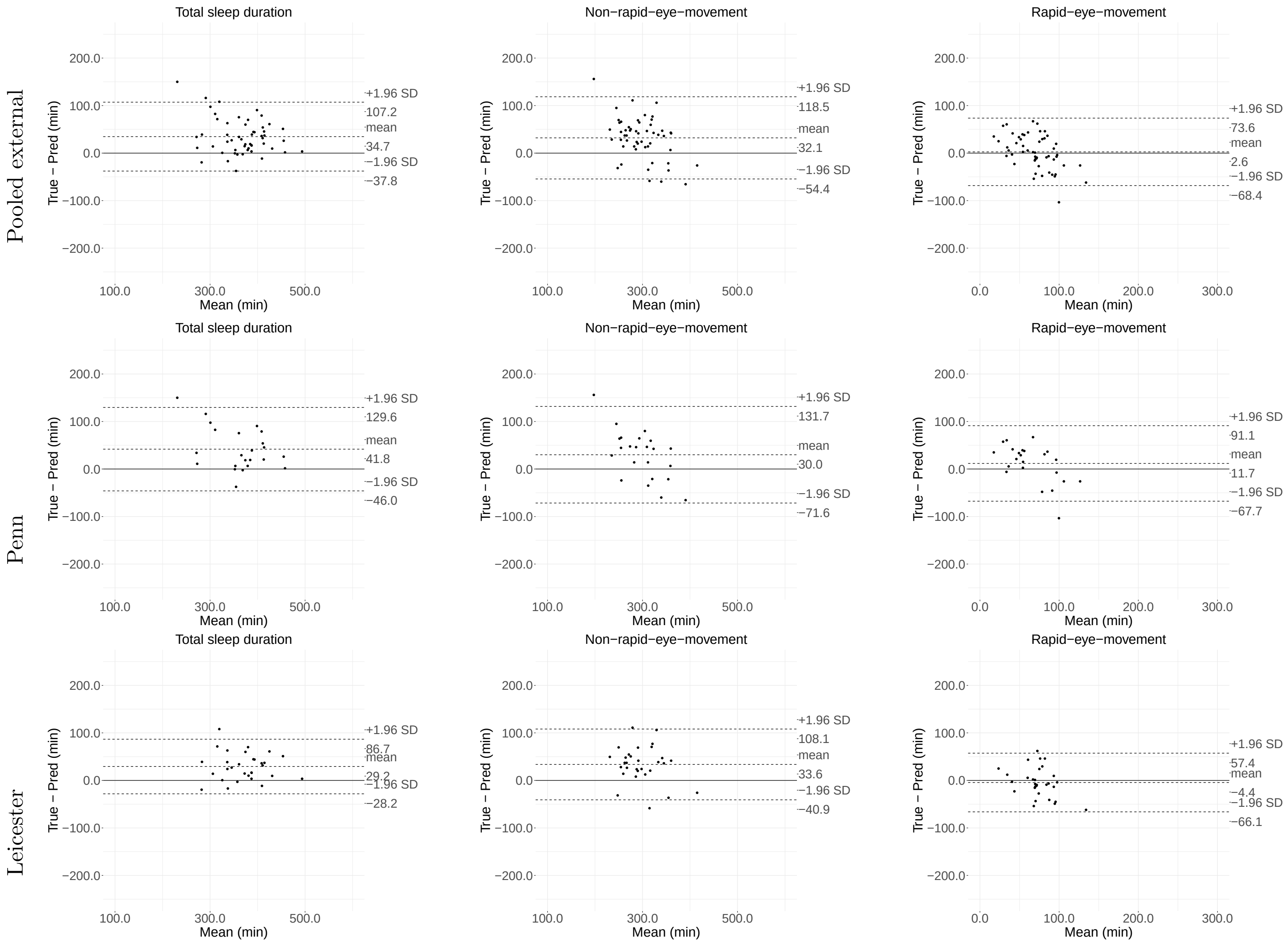

Figure 6: **Agreement assessment via Bland-Altman plots for external validation: total sleep duration, wake after sleep onset (WASO), non-rapid-eye-movement sleep (NREM), and rapid-eye-movement sleep (REM).**

medRxiv preprint doi: https://doi.org/10.1101/2023.07.07.23292251; this version posted July 8, 2023. The copyright holder for this preprint (which was not certified by peer review) is the author/funder, who has granted medRxiv a license to display the preprint in perpetuity.
It is made available under a CC-BY 4.0 International license .

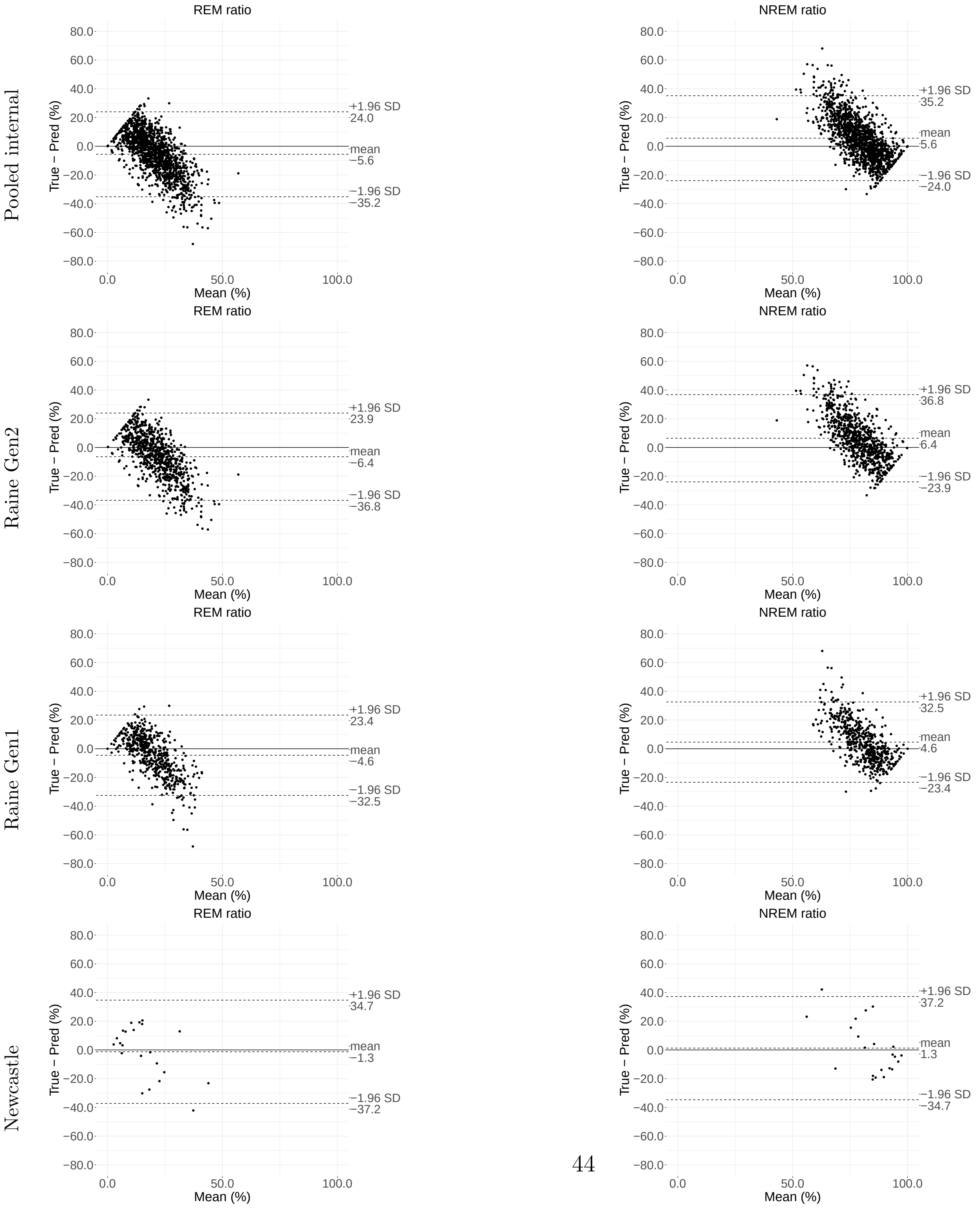

Figure 7: **Agreement assessment via Bland-Altman plots for internal validation: non-rapid-eye-movement sleep (NREM) ratio, and rapid-eye-movement sleep (REM) ratio.**

medRxiv preprint doi: https://doi.org/10.1101/2023.07.07.23292251; this version posted July 8, 2023. The copyright holder for this preprint
(which was not certified by peer review) is the author/funder, who has granted medRxiv a license to display the preprint in perpetuity.
It is made available under a CC-BY 4.0 International license .

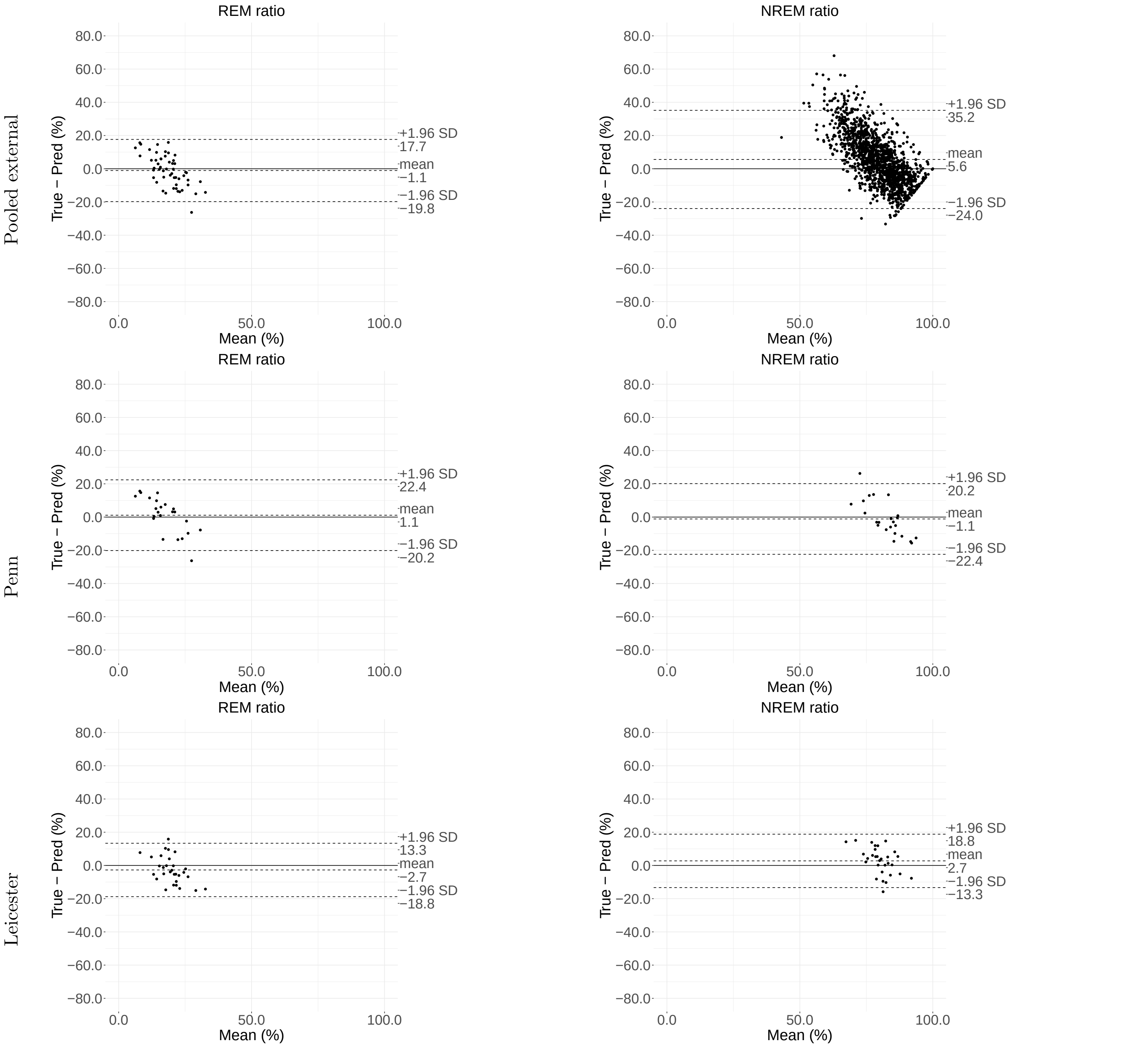

Figure 8: **Agreement assessment via Bland-Altman plots for external validation: non-rapid-eye-movement sleep (NREM) ratio, and rapid-eye-movement sleep (REM) ratio.**

medRxiv preprint doi: https://doi.org/10.1101/2023.07.07.23292251; this version posted July 8, 2023. The copyright holder for this preprint (which was not certified by peer review) is the author/funder, who has granted medRxiv a license to display the preprint in perpetuity.
It is made available under a CC-BY 4.0 International license .

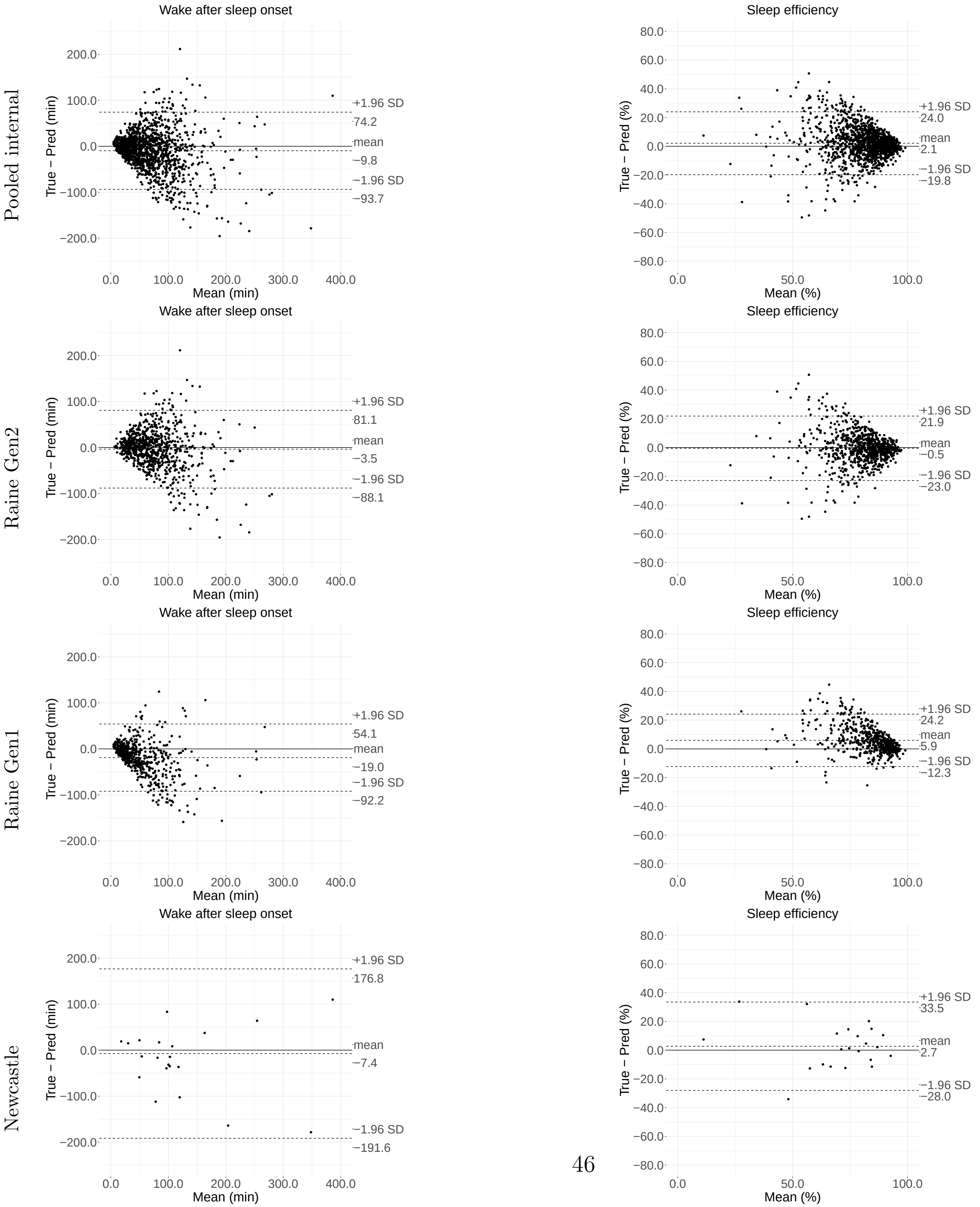

Figure 9: **Agreement assessment via Bland-Altman plots for internal validation: wake after sleep onset (WASO), and sleep efficiency (SE).**

medRxiv preprint doi: https://doi.org/10.1101/2023.07.07.23292251; this version posted July 8, 2023. The copyright holder for this preprint (which was not certified by peer review) is the author/funder, who has granted medRxiv a license to display the preprint in perpetuity.
It is made available under a CC-BY 4.0 International license .

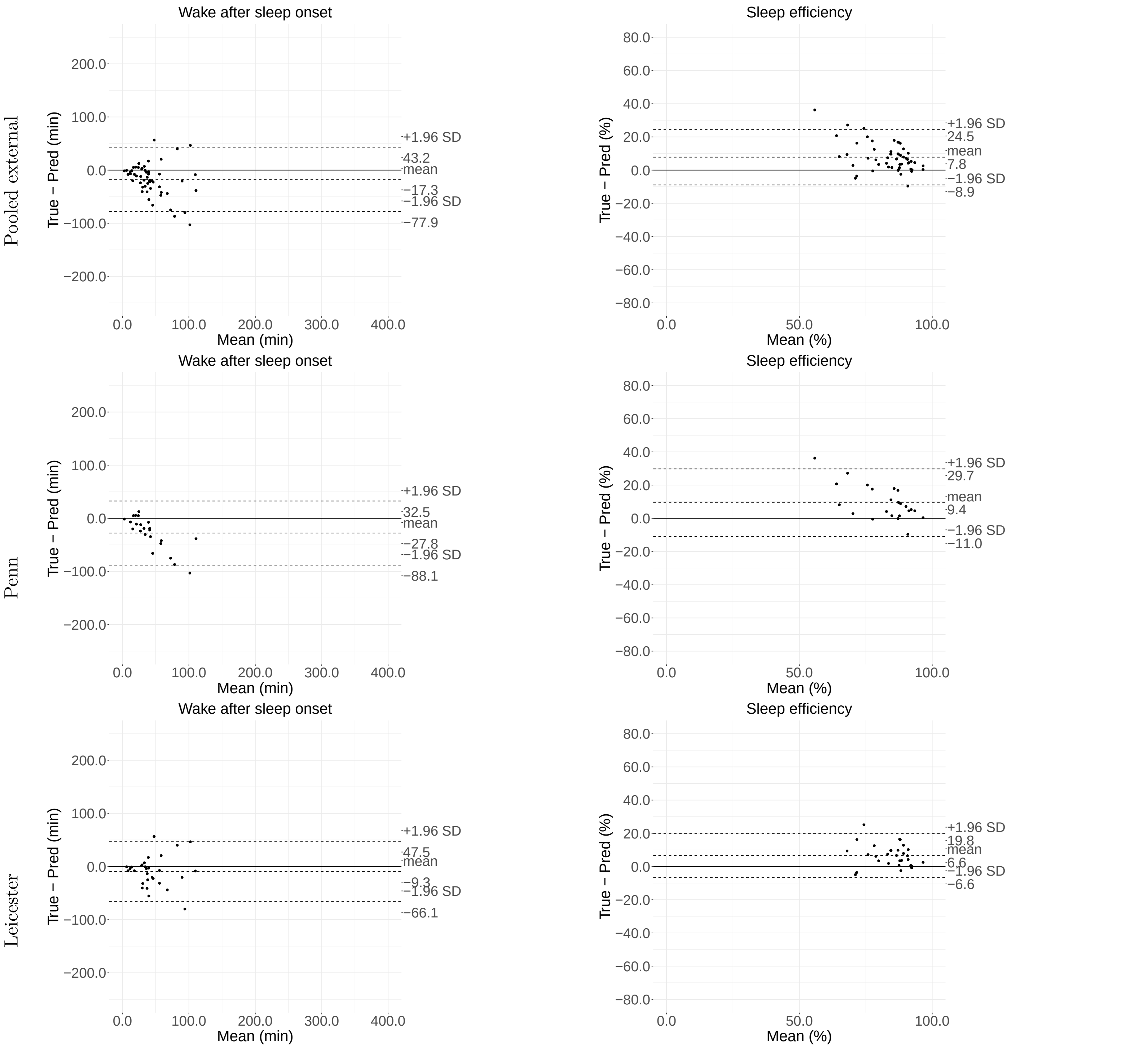

Figure 10: **Agreement assessment via Bland-Altman plots for internal validation: wake after sleep onset (WASO), and sleep efficiency (SE).**

medRxiv preprint doi: https://doi.org/10.1101/2023.07.07.23292251; this version posted July 8, 2023. The copyright holder for this preprint
(which was not certified by peer review) is the author/funder, who has granted medRxiv a license to display the preprint in perpetuity.
It is made available under a CC-BY 4.0 International license .

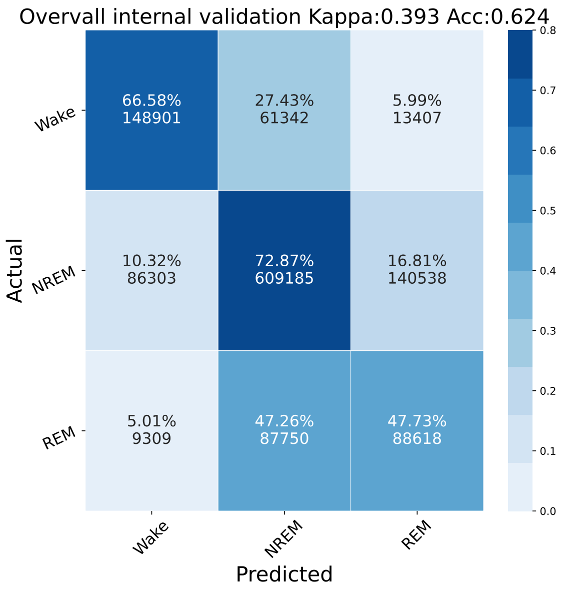

(a) Overall internal validation

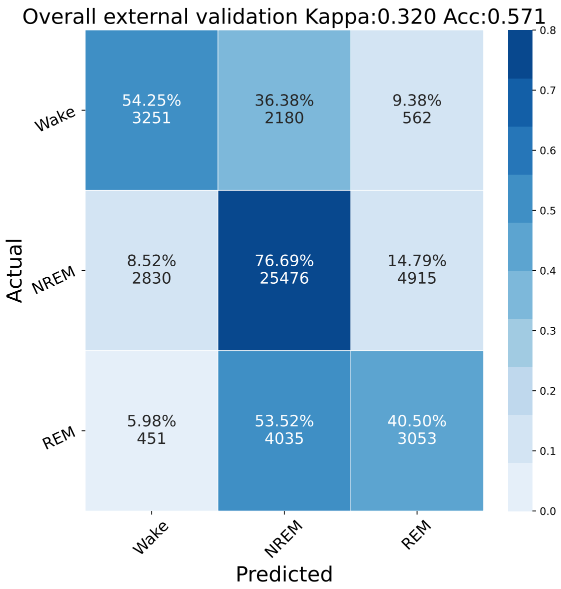

(b) Overall external validation

Figure 11: **Three class classification (wake/REM/NREM) confusion matrix**: epoch-to-epoch Kappa and balanced accuracies are shown. The number of predictions and proportion ratios are shown for each pair of ground-truth and prediction class. REM: rapid-eye-movement sleep; NREM: non-rapid-eye-movement sleep.

medRxiv preprint doi: https://doi.org/10.1101/2023.07.07.23292251; this version posted July 8, 2023. The copyright holder for this preprint (which was not certified by peer review) is the author/funder, who has granted medRxiv a license to display the preprint in perpetuity.
It is made available under a CC-BY 4.0 International license .

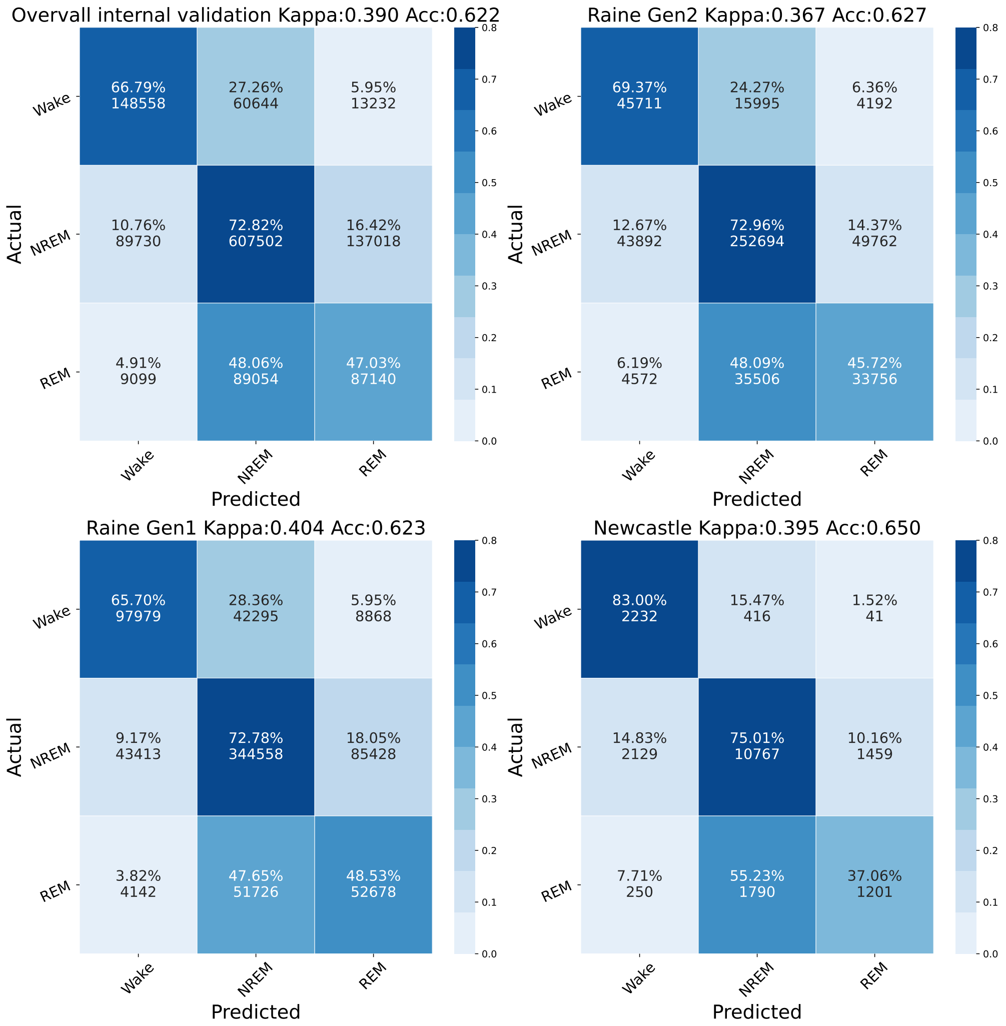

Figure 12: **Three-class sleep staging (wake/REM/NREM) for internal validation: epoch-to-epoch Kappa and balanced accuracies are shown.** The number of predictions and proportion ratios are shown for each pair of ground-truth and prediction class. REM: rapid-eye-movement sleep; NREM: non-rapid-eye-movement sleep.

medRxiv preprint doi: https://doi.org/10.1101/2023.07.07.23292251; this version posted July 8, 2023. The copyright holder for this preprint (which was not certified by peer review) is the author/funder, who has granted medRxiv a license to display the preprint in perpetuity.
It is made available under a CC-BY 4.0 International license .

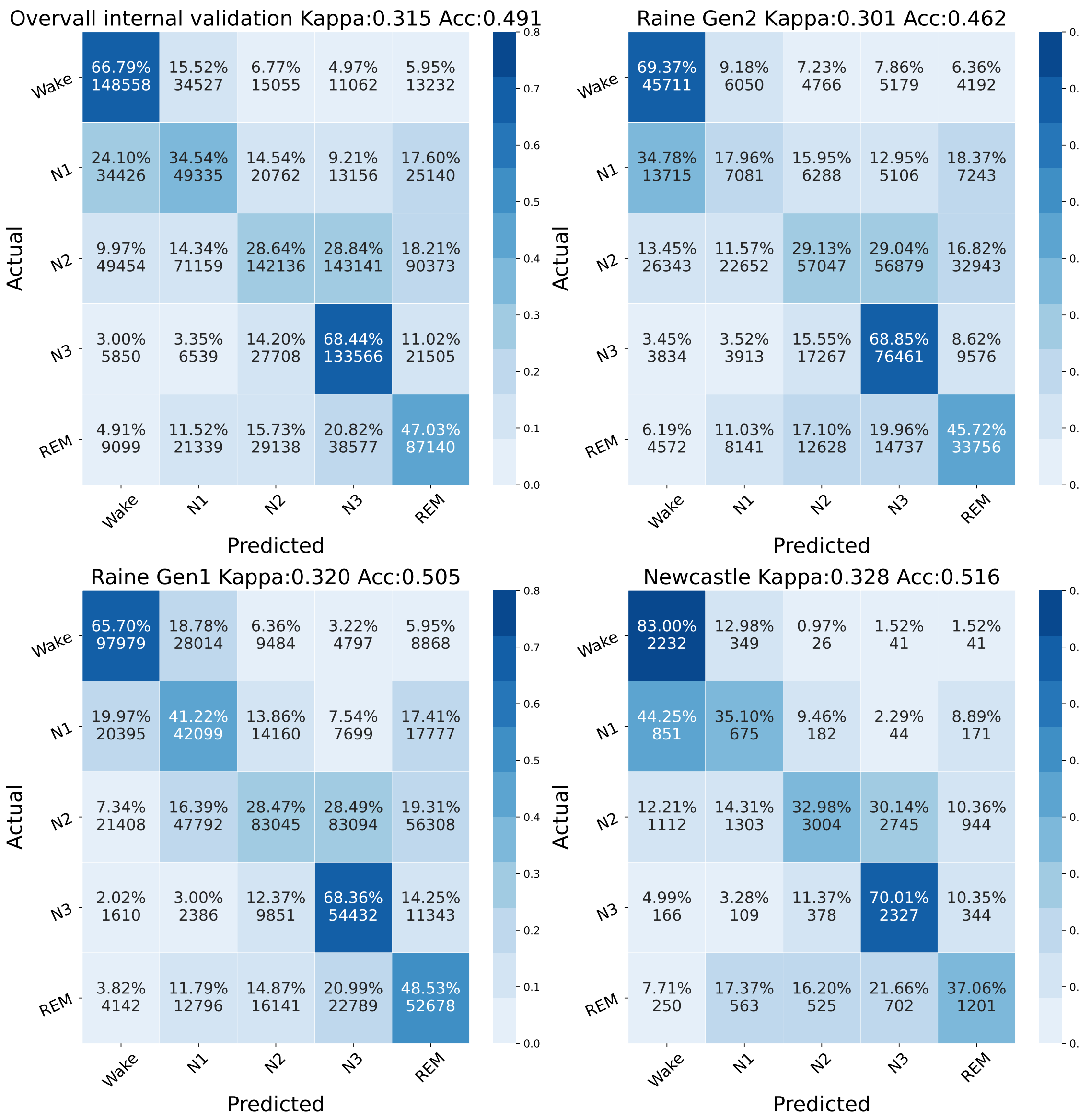

Figure 13: **Five-class sleep staging (wake/REM/N1/N2/N3) for internal validation: epoch-to-epoch kappa and balanced accuracies are shown.** The number of predictions and proportion ratios are shown for each pair of ground-truth and prediction class. REM: rapid-eye-movement sleep, N1, N2, N3: non-rapid-eye-movement sleep 1, 2, 3.

medRxiv preprint doi: https://doi.org/10.1101/2023.07.07.23292251; this version posted July 8, 2023. The copyright holder for this preprint (which was not certified by peer review) is the author/funder, who has granted medRxiv a license to display the preprint in perpetuity.
It is made available under a CC-BY 4.0 International license .

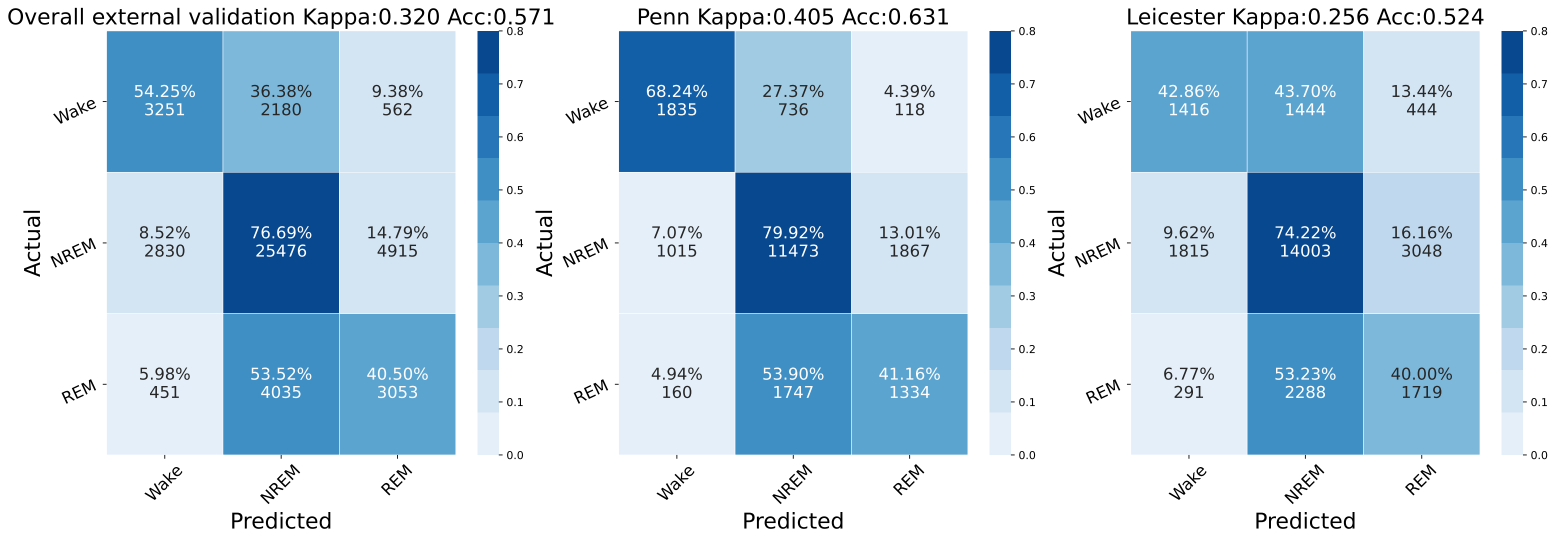

Figure 14: **Three-class sleep staging (wake/REM/NREM) for external validation: epoch-to-epoch kappa and balanced accuracies are shown.** The number of predictions and proportion ratios are shown for each pair of ground-truth and prediction class. REM: rapid-eye-movement sleep; NREM: non-rapid-eye-movement sleep.

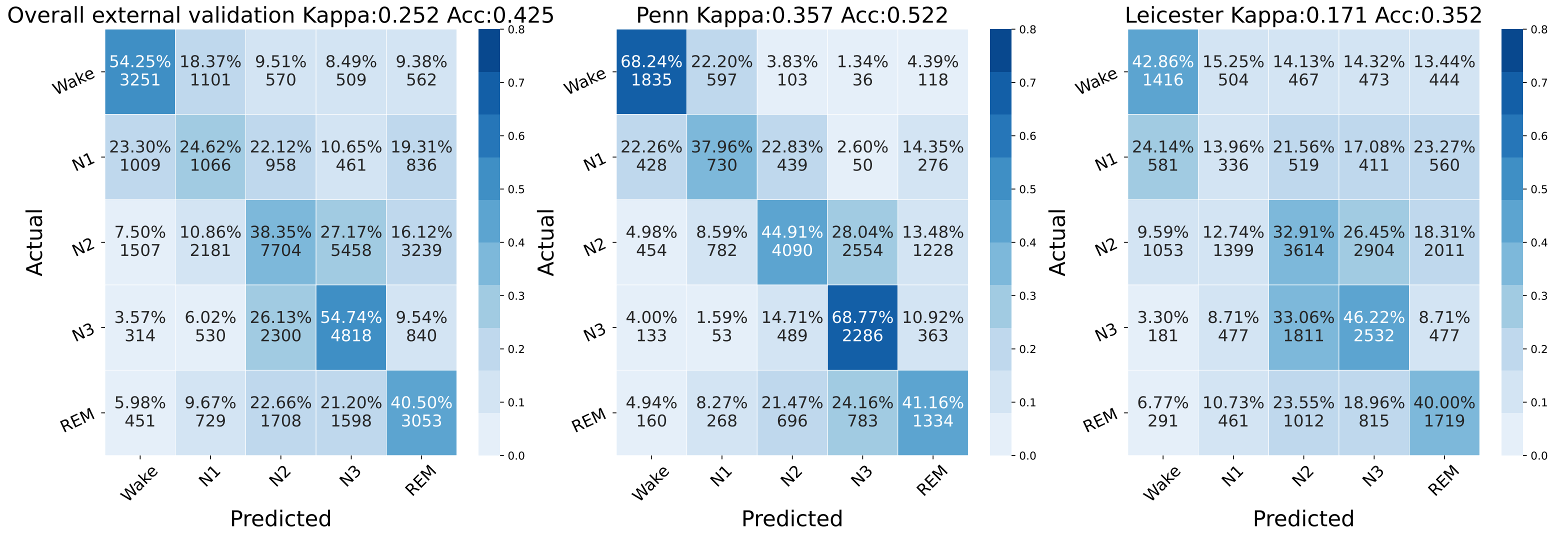

Figure 15: **Five-class sleep staging (wake/REM/N1/N2/N3) for external validation: epoch-to-epoch kappa and balanced accuracies are shown.** The number of predictions and proportion ratios are shown for each pair of ground-truth and prediction class. REM: rapid-eye-movement sleep, N1, N2, N3: non-rapid-eye-movement sleep 1, 2, 3.

medRxiv preprint doi: https://doi.org/10.1101/2023.07.07.23292251; this version posted July 8, 2023. The copyright holder for this preprint (which was not certified by peer review) is the author/funder, who has granted medRxiv a license to display the preprint in perpetuity.
It is made available under a CC-BY 4.0 International license .

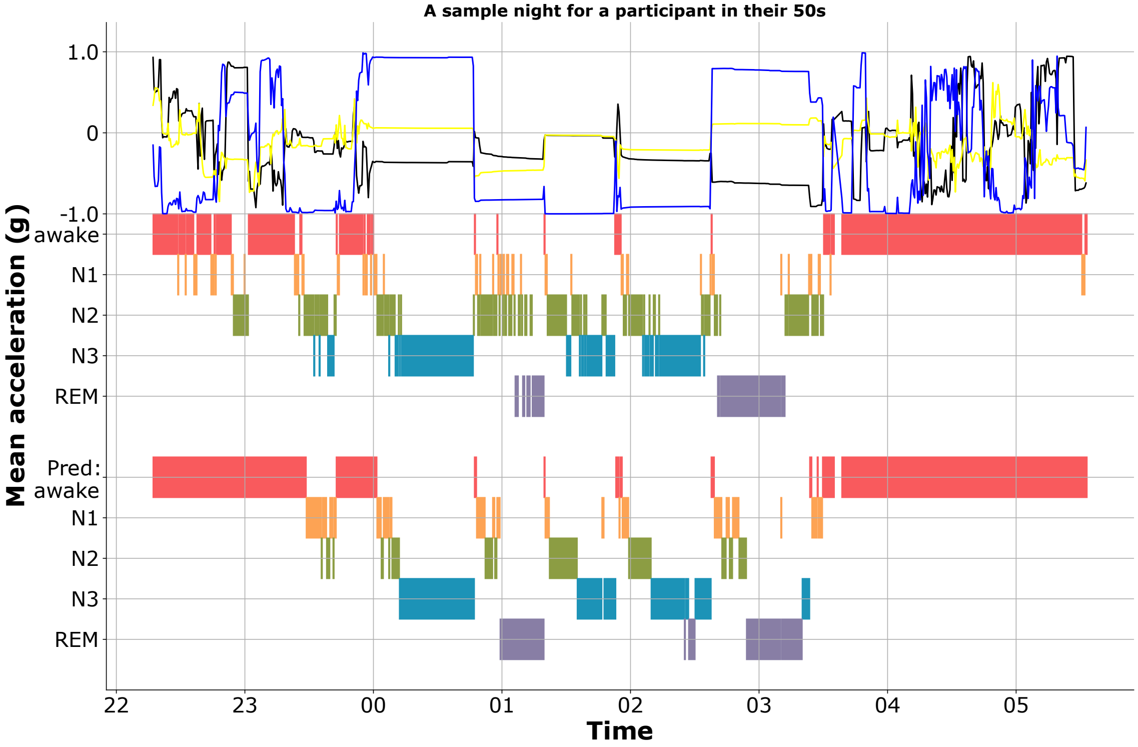

Figure 16: **A sample actigram, hypnogram ground truth and prediction for a participant whose sleep stages are well captured:** the **top** hypnogram is the ground-truth and the **bottom** hypnogram is the prediction generated by SleepNet based on the actigram. REM: rapid-eye-movement sleep, N1, N2, N3: non-rapid-eye-movement sleep 1, 2, 3.

medRxiv preprint doi: https://doi.org/10.1101/2023.07.07.23292251; this version posted July 8, 2023. The copyright holder for this preprint (which was not certified by peer review) is the author/funder, who has granted medRxiv a license to display the preprint in perpetuity.
It is made available under a CC-BY 4.0 International license .

798 *8.3. Additional results on the sleep variations for the UK Biobank participants*

medRxiv preprint doi: https://doi.org/10.1101/2023.07.07.23292251; this version posted July 8, 2023. The copyright holder for this preprint (which was not certified by peer review) is the author/funder, who has granted medRxiv a license to display the preprint in perpetuity.
It is made available under a CC-BY 4.0 International license .

Figure 17: **Participant flow diagram for the analysis of sleep and all-cause mortality in the UK Biobank.** TDI: Townsend deprivation index, BMI: body mass index, SR_health: self-reported overall health, SR_insomnia: self-reported insomnia symptoms, CVD: Cardiovascular disease.

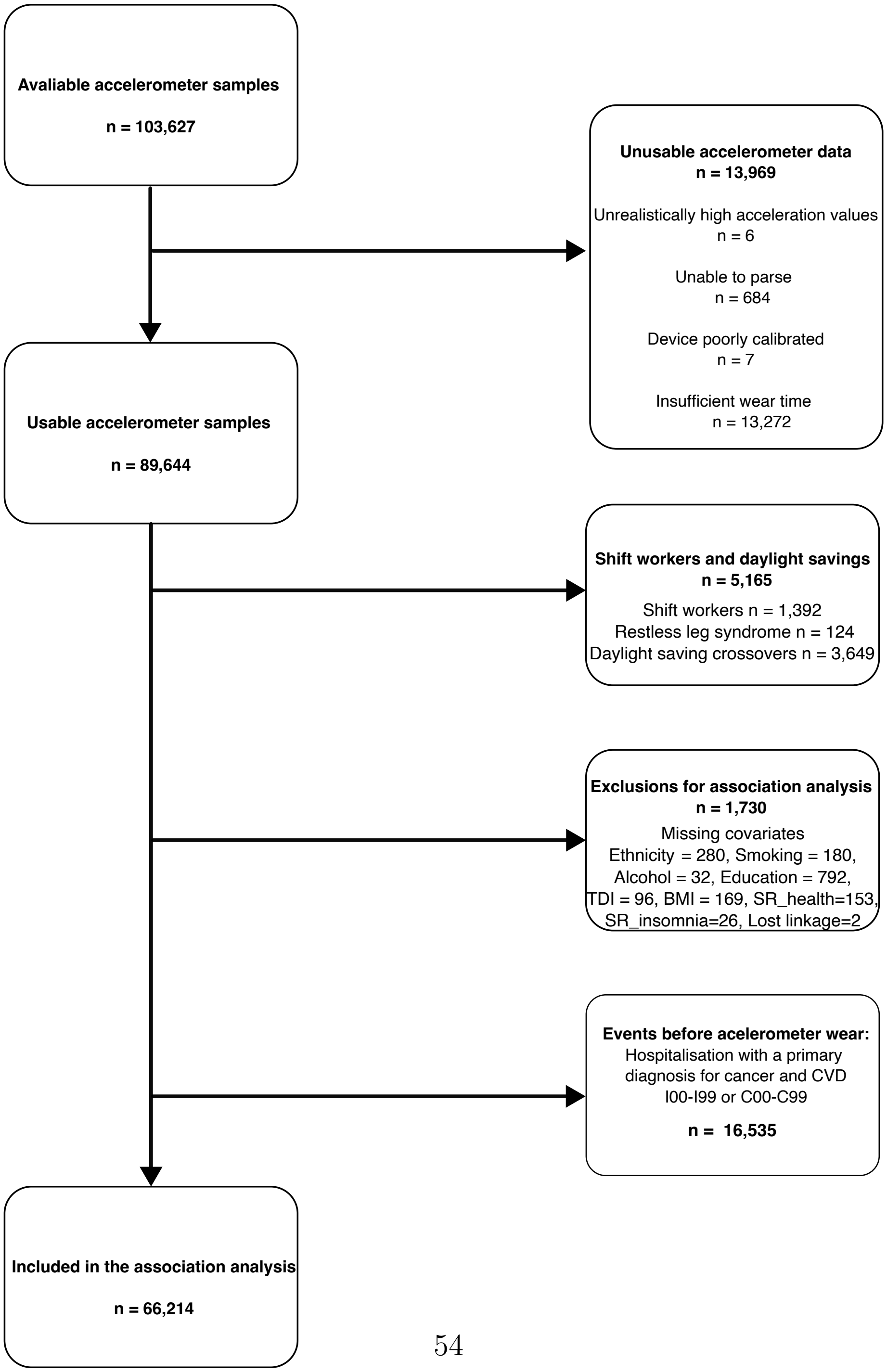

medRxiv preprint doi: https://doi.org/10.1101/2023.07.07.23292251; this version posted July 8, 2023. The copyright holder for this preprint (which was not certified by peer review) is the author/funder, who has granted medRxiv a license to display the preprint in perpetuity.
It is made available under a CC-BY 4.0 International license .

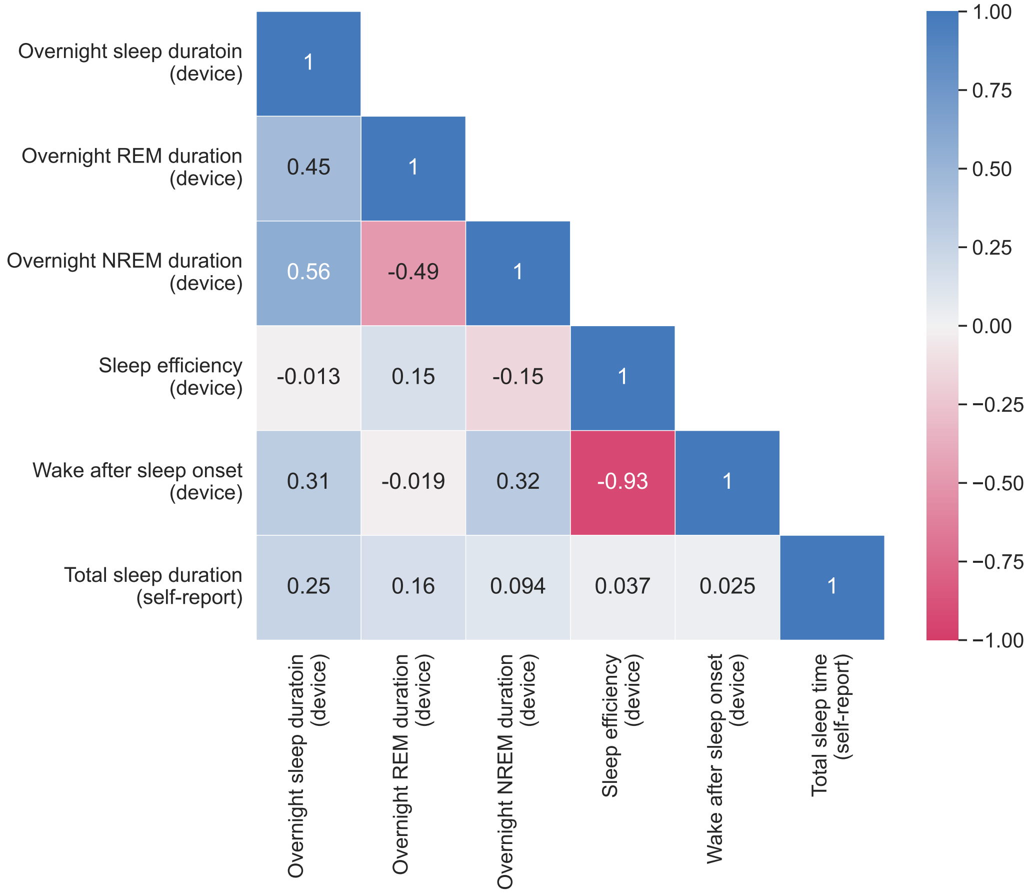

Figure 18: **Correlation matrix for device-measured and self-reported sleep parameters on the UK Biobank.** The self-reported total sleep duration was obtained via questionnaire at baseline assessment in the UK Biobank. REM: rapid-eye-movement sleep, NREM: non-rapid-eye-movement sleep.

medRxiv preprint doi: https://doi.org/10.1101/2023.07.07.23292251; this version posted July 8, 2023. The copyright holder for this preprint
(which was not certified by peer review) is the author/funder, who has granted medRxiv a license to display the preprint in perpetuity.
It is made available under a CC-BY 4.0 International license .

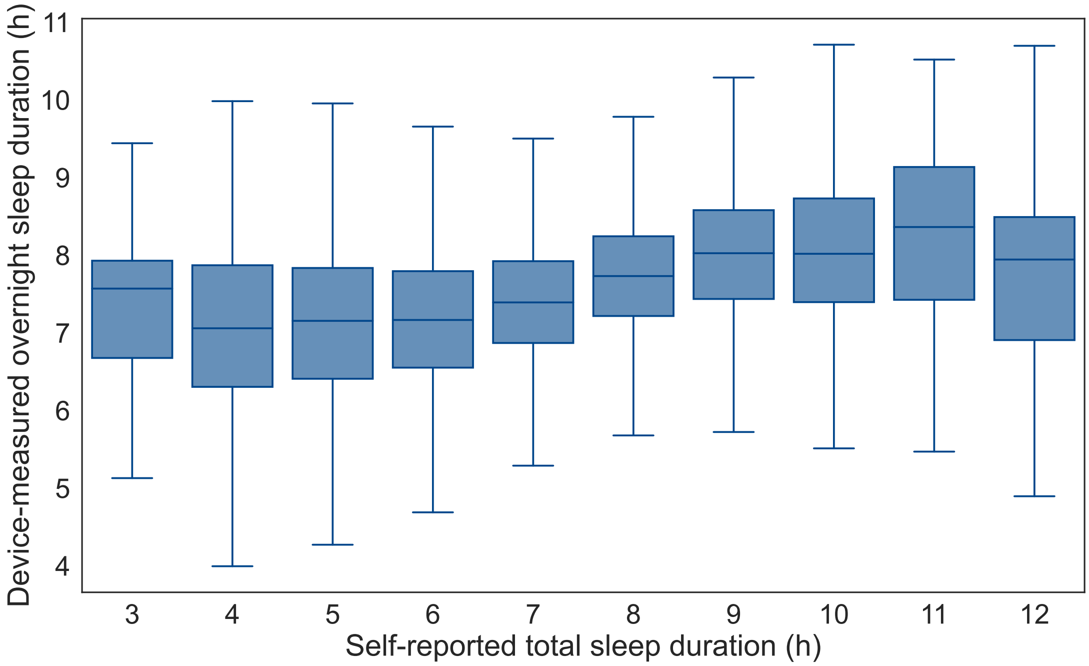

Figure 19: **Box plots showing the distributions of device-measured overnight sleep duration against self-reported total sleep duration.** The box whiskers reflect the lowest and highest data points that are 1.5 times of the inter-quartile-range from the median.

medRxiv preprint doi: https://doi.org/10.1101/2023.07.07.23292251; this version posted July 8, 2023. The copyright holder for this preprint (which was not certified by peer review) is the author/funder, who has granted medRxiv a license to display the preprint in perpetuity.
It is made available under a CC-BY 4.0 International license .

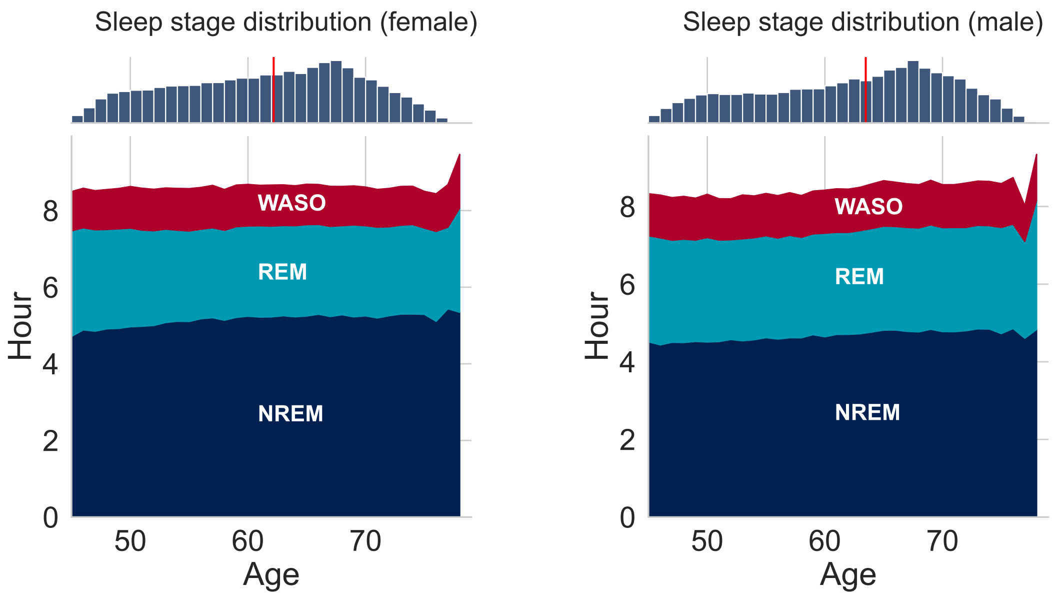

Figure 20: **The average device-measured sleep stage distribution with respect to age for both females (left) and males (right) on the UK Biobank.** The histograms on the top show the age distribution for the participants. The red vertical line denotes the median age for each sex. WASO: wake after sleep onset; REM: rapid-eye-movement sleep; NREM: non-rapid-eye-movement sleep.

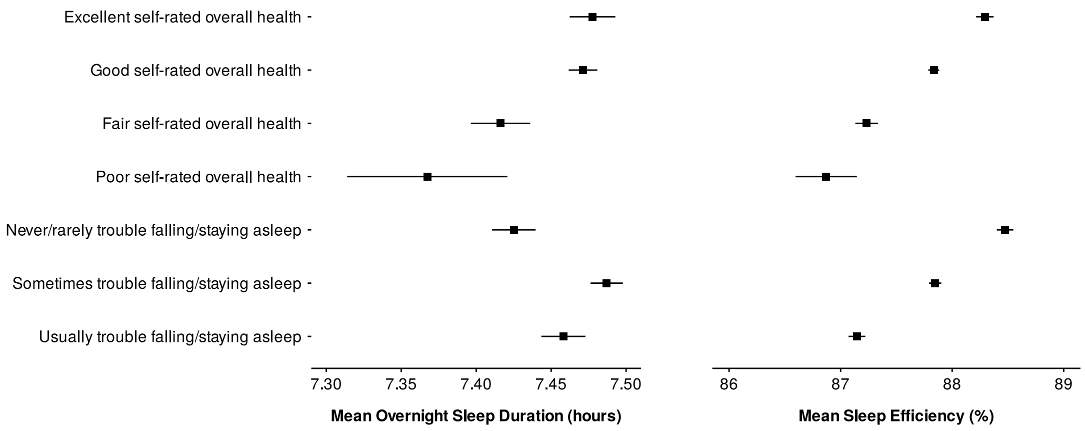

Figure 21: **Adjusted marginal mean (95% confidence interval) device-measured mean overnight sleep duration and mean sleep efficiency by self-reported overall health status and insomnia history in the UK Biobank.** Mean overnight sleep duration and sleep efficiency were adjusted for age and sex.

medRxiv preprint doi: https://doi.org/10.1101/2023.07.07.23292251; this version posted July 8, 2023. The copyright holder for this preprint (which was not certified by peer review) is the author/funder, who has granted medRxiv a license to display the preprint in perpetuity.
It is made available under a CC-BY 4.0 International license .

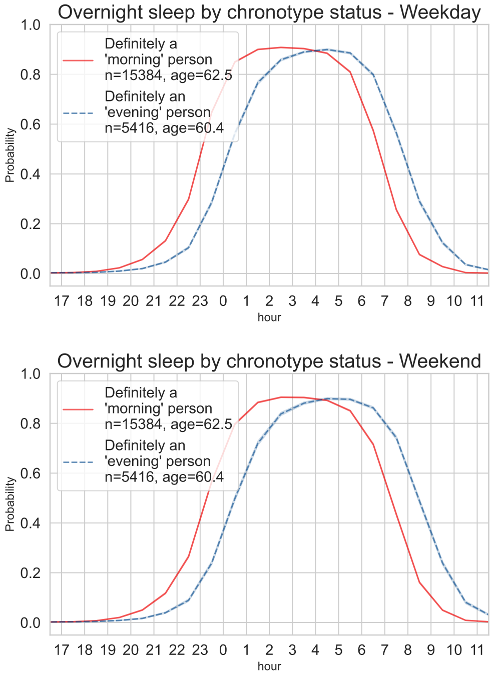

(a) Chronotype

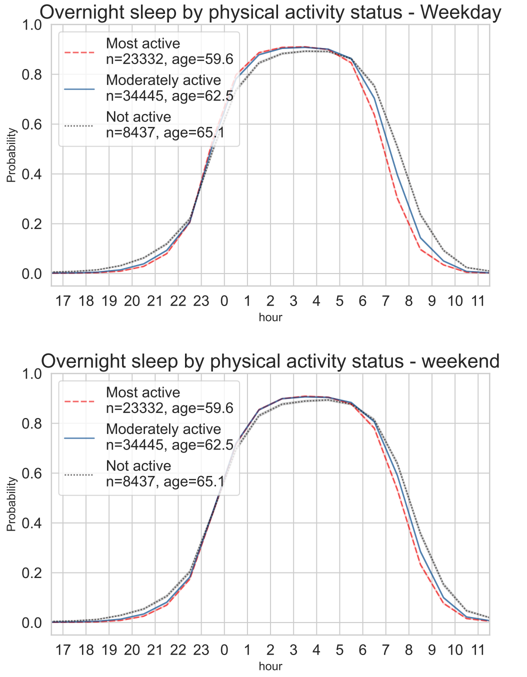

(b) Physical activity

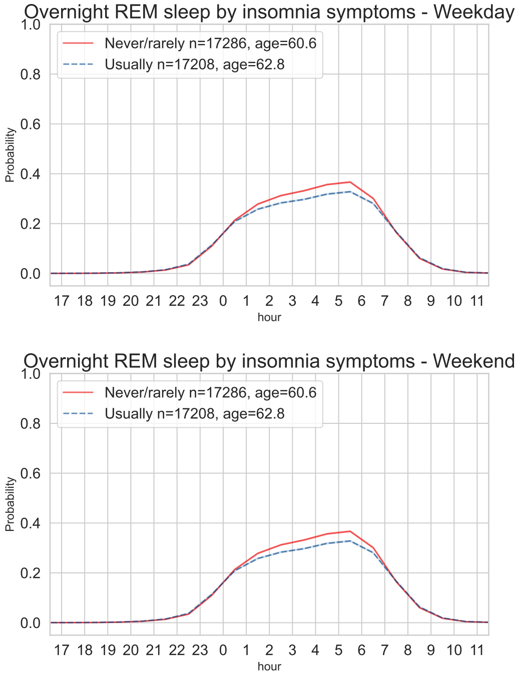

(c) Insomnia symptoms REM

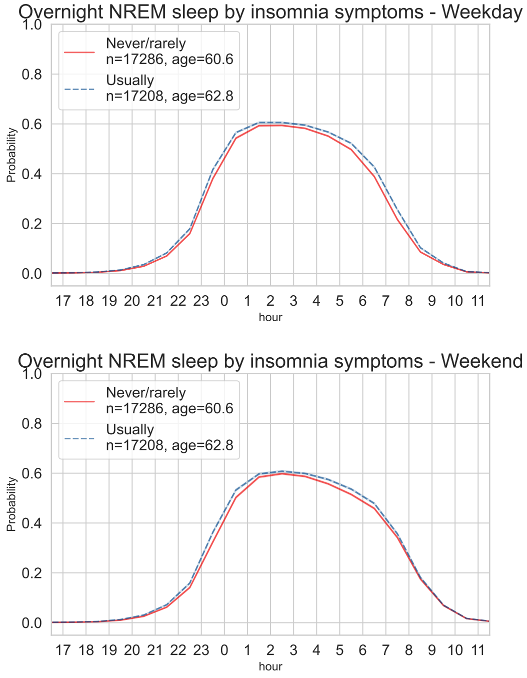

(d) Insomnia symptoms NREM

Figure 22: **Device-measured sleep probability trajectories throughout the day for the UK Biobank participants (weekday vs weekend).** Top: variations of the average overnight sleep probability for the participants with self-reported "morning" and "evening" chronotype (a) and the overnight sleep distributions across thirds of device-measured physical activity level (b). Bottom: variations of the average REM (c) and NREM (d) probability in participants with a history of self-reported insomnia symptoms versus those without. Rapid-eye-movement sleep (REM), and non-rapid-eye-movement sleep (NREM). Areas of squares represent the inverse of the variance of the log risk. And the I bars denote the 95% confidence interval for the floated risks.

medRxiv preprint doi: https://doi.org/10.1101/2023.07.07.23292251; this version posted July 8, 2023. The copyright holder for this preprint (which was not certified by peer review) is the author/funder, who has granted medRxiv a license to display the preprint in perpetuity.
It is made available under a CC-BY 4.0 International license .

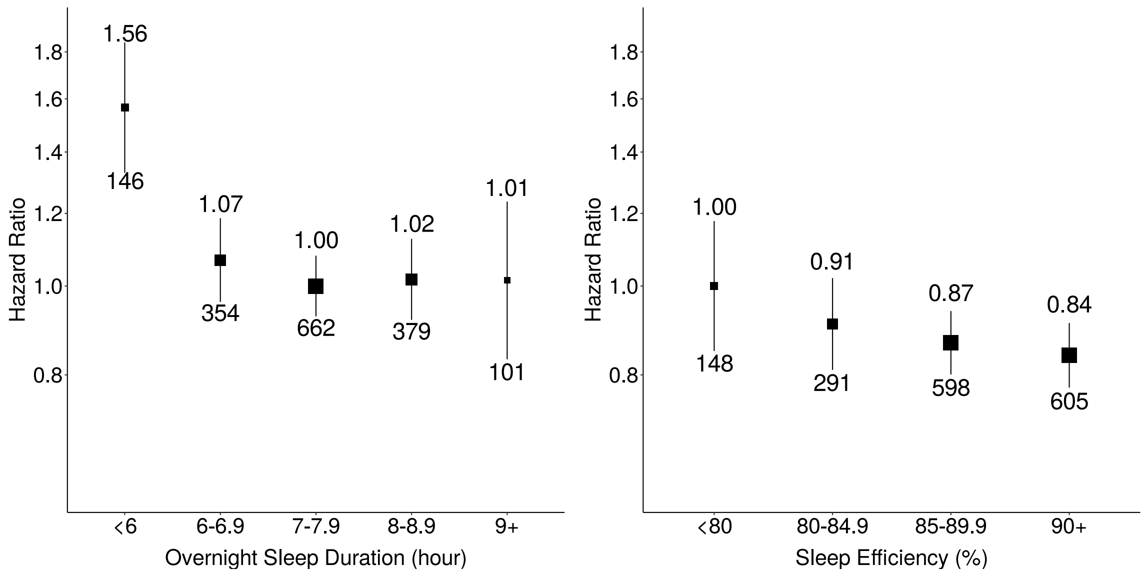

(a) (b)

Figure 23: **Associations of overnight sleep duration (a) and sleep efficiency (b) with all-cause mortality.** The model used 1,642 events among 62,214 participants. We used age as the timescale and adjusted for sex, ethnicity, Townsend Deprivation Index of baseline address (split by quarter in the study population), educational qualifications, smoking status, alcohol consumption (Never, <3 times/week, 3+ times/week), overall activity (measured in milli-gravity units). Areas of squares represent the inverse of the variance of the log risk. The I bars denote the 95% confidence interval for the floated risks.

medRxiv preprint doi: https://doi.org/10.1101/2023.07.07.23292251; this version posted July 8, 2023. The copyright holder for this preprint (which was not certified by peer review) is the author/funder, who has granted medRxiv a license to display the preprint in perpetuity.
It is made available under a CC-BY 4.0 International license .

8.3.1. Models additionally adjusted for body mass index

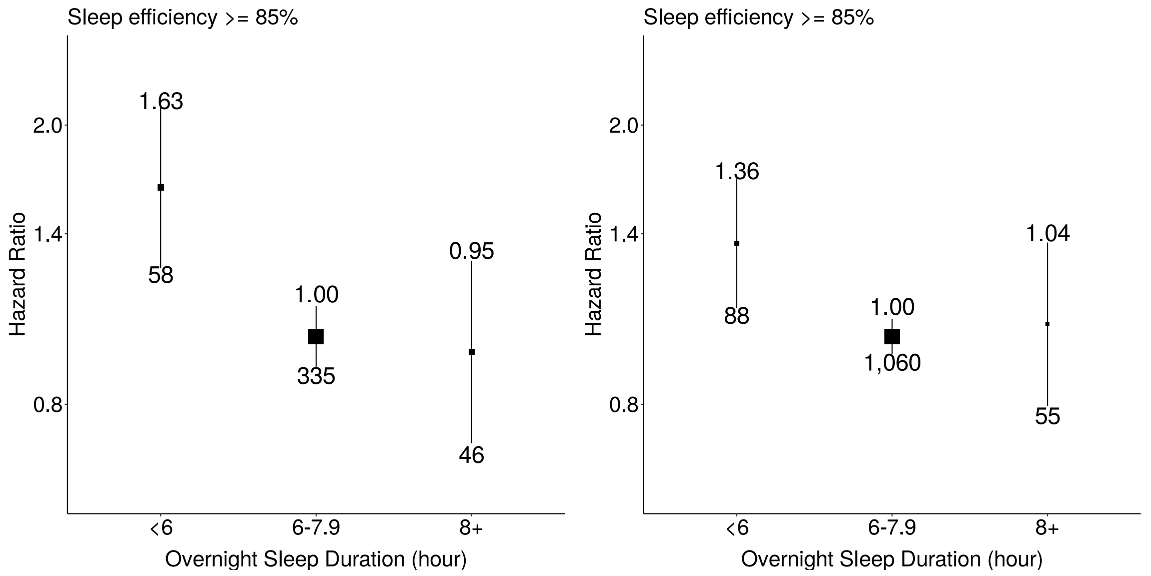

(a) (b)

Figure 24: **Associations of overnight sleep duration with all-cause mortality for groups with low and high sleep efficiency additionally adjusted for body mass index.** The model used 1,642 events among 62,214 participants. We used age as the timescale and adjusted for sex, ethnicity, Townsend Deprivation Index of baseline address (split by quarter in the study population), educational qualifications, smoking status, alcohol consumption (Never, <3 times/week, 3+ times/week), overall activity (measured in milli-gravity units). Areas of squares represent the inverse of the variance of the log risk. The I bars denote the 95% confidence interval for the floated risks.

medRxiv preprint doi: https://doi.org/10.1101/2023.07.07.23292251; this version posted July 8, 2023. The copyright holder for this preprint (which was not certified by peer review) is the author/funder, who has granted medRxiv a license to display the preprint in perpetuity.
It is made available under a CC-BY 4.0 International license .

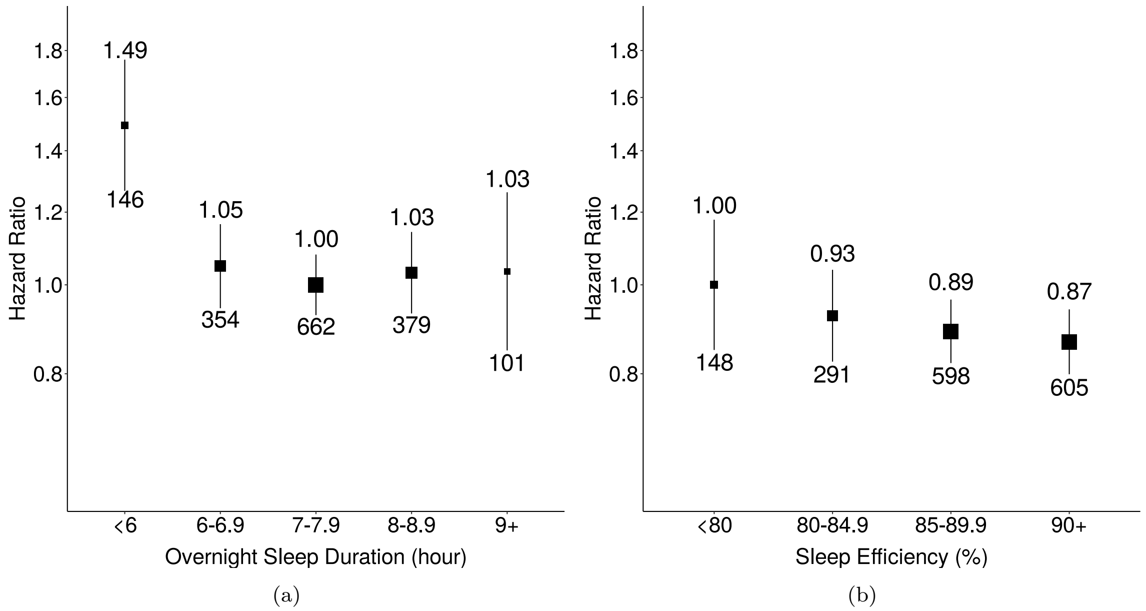

Figure 25: **Associations of overnight sleep duration (a) and sleep efficiency (b) with all-cause mortality additionally adjusted for body mass index.** The model used 1,642 events among 62,214 participants. We used age as the timescale and adjusted for sex, ethnicity, Townsend Deprivation Index of baseline address (split by quarter in the study population), educational qualifications, smoking status, alcohol consumption (Never, <3 times/week, 3+ times/week), overall activity (measured in milli-gravity units), and body mass index. Areas of squares represent the inverse of the variance of the log risk. The I bars denote the 95% confidence interval for the floated risks.

medRxiv preprint doi: https://doi.org/10.1101/2023.07.07.23292251; this version posted July 8, 2023. The copyright holder for this preprint (which was not certified by peer review) is the author/funder, who has granted medRxiv a license to display the preprint in perpetuity.
It is made available under a CC-BY 4.0 International license .

8.3.2. Sensitivity analysis for overnight sleep duration

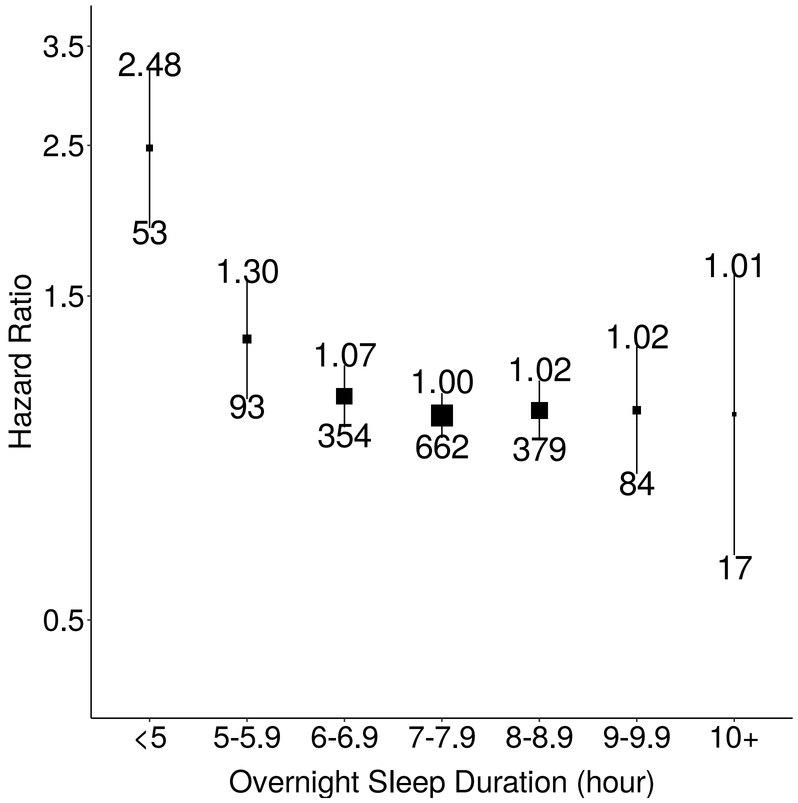

Figure 26: **Associations of device-measured overnight sleep duration and all-cause mortality with greater granularity.** The model used 1,642 events among 62,214 participants. We used age as the timescale and adjusted for sex, ethnicity, Townsend Deprivation Index of baseline address (split by quarter in the study population), educational qualifications, smoking status, alcohol consumption (Never, <3 times/week, 3+ times/week), and overall activity (measured in milli-gravity units). Areas of squares represent the inverse of the variance of the log risk. The I bars denote the 95% confidence interval for the floated risks.

medRxiv preprint doi: https://doi.org/10.1101/2023.07.07.23292251; this version posted July 8, 2023. The copyright holder for this preprint (which was not certified by peer review) is the author/funder, who has granted medRxiv a license to display the preprint in perpetuity.
It is made available under a CC-BY 4.0 International license .

References

[1] Aiden Doherty et al. "Large scale population assessment of physical activity using wrist worn accelerometers: the UK biobank study". In: *PloS One* 12.2 (2017), e0169649.

[2] Leon Straker et al. "Cohort profile: the Western Australian pregnancy cohort (Raine) study–Generation 2". In: *International Journal of Epidemiology* 46.5 (2017), 1384–1385j.

[3] Vincent van Hees, Sarah Charman, and Kirstie Anderson. *Newcastle polysomnography and accelerometer data*. Version 1.0. Zenodo, Jan. 2018. DOI: 10.5281/zenodo.1160410. URL: https://doi.org/10.5281/zenodo.1160410.

[4] Tatiana Plekhanova et al. "Validation of an automated sleep detection algorithm using data from multiple accelerometer brands". In: *Journal of Sleep Research* (2022).

[5] Enda M Byrne et al. "Genetic correlation analysis suggests association between increased self-reported sleep duration in adults and schizophrenia and type 2 diabetes". In: *Sleep* 39.10 (2016), pp. 1853–1857.

[6] Manon L Dontje, Peter Eastwood, and Leon Straker. "Western Australian pregnancy cohort (Raine) study: generation 1". In: *BMJ open* 9.5 (2019), e026276.

[7] Cathie Sudlow et al. "UK biobank: an open access resource for identifying the causes of a wide range of complex diseases of middle and old age". In: *PLoS Medicine* 12.3 (2015), e1001779.

[8] Hang Yuan et al. "Self-supervised Learning for Human Activity Recognition Using 700,000 Person-days of Wearable Data". In: *arXiv preprint arXiv:2206.02909* (2022).

[9] Kaiming He et al. "Identity mappings in deep residual networks". In: *European Conference on Computer Vision*. Springer. 2016, pp. 630–645.

[10] Diederik P Kingma and Jimmy Ba. "Adam: A method for stochastic optimization". In: *arXiv preprint arXiv:1412.6980* (2014).

medRxiv preprint doi: https://doi.org/10.1101/2023.07.07.23292251; this version posted July 8, 2023. The copyright holder for this preprint (which was not certified by peer review) is the author/funder, who has granted medRxiv a license to display the preprint in perpetuity.
It is made available under a CC-BY 4.0 International license .

[11] Zhiheng Huang, Wei Xu, and Kai Yu. "Bidirectional LSTM-CRF models for sequence tagging". In: *arXiv preprint arXiv:1508.01991* (2015).

[12] Kalaivani Sundararajan et al. "Sleep classification from wrist-worn accelerometer data using random forests". In: *Scientific Reports* 11.1 (2021), pp. 1–10.

[13] Rosemary Walmsley et al. "Reallocation of time between device-measured movement behaviours and risk of incident cardiovascular disease". In: *British Journal of Sports Medicine* 56.18 (2022), pp. 1008–1017.

[14] Max Hirshkowitz et al. "National Sleep Foundation's updated sleep duration recommendations". In: *Sleep health* 1.4 (2015), pp. 233–243.

[15] Bin Yan et al. "Objective sleep efficiency predicts cardiovascular disease in a community population: the sleep heart health study". In: *Journal of the American Heart Association* 10.7 (2021), e016201.

[16] Douglas F Easton, Julian Peto, and Abdel GAG Babiker. "Floating absolute risk: an alternative to relative risk in survival and case-control analysis avoiding an arbitrary reference group". In: *Statistics in Medicine* 10.7 (1991), pp. 1025–1035.

[17] Martyn Plummer and Bendix Carstensen. "Lexis: An R Class for Epidemiological Studies with Long-Term Follow-Up". In: *Journal of Statistical Software* 38.5 (2011), pp. 1–12. URL: `https://www.jstatsoft.org/v38/i05/`.

[18] Martyn Plummer. "Improved estimates of floating absolute risk". In: *Statistics in Medicine* 23.1 (2004), pp. 93–104.

[19] Terry K Koo and Mae Y Li. "A guideline of selecting and reporting intraclass correlation coefficients for reliability research". In: *Journal of Chiropractic Medicine* 15.2 (2016), pp. 155–163.
